# Circ_005077 accelerates myocardial lipotoxicity induced by high-fat diet via CyPA/p47PHOX mediated ferroptosis

**DOI:** 10.1186/s12933-024-02204-3

**Published:** 2024-04-15

**Authors:** Xinzhu Ni, Lian Duan, Yandong Bao, Jinyang Li, Xiaowen Zhang, Dalin Jia, Nan Wu

**Affiliations:** 1 Department of Cardiology, The First Affiliated Hospital of China Medical University, Shenyang, 110001 Liaoning P.R. China; 2 Department of Geriatric Cardiology, The First Affiliated Hospital of China Medical University, Shenyang, Liaoning PR China; 3 Medical Research Center, Shengjing Hospital of China Medical University, Shenyang, Liaoning PR China; 4 Department of Central Laboratory, The First Affiliated Hospital of China Medical University, Shenyang, Liaoning PR China

**Keywords:** High-fat diet, Myocardial lipotoxicity, Ferroptosis, Cyclophilin A

## Abstract

The long-term high-fat diet (HFD) can cause myocardial lipotoxicity, which is characterized pathologically by myocardial hypertrophy, fibrosis, and remodeling and clinically by cardiac dysfunction and heart failure in patients with obesity and diabetes. Circular RNAs (circRNAs), a novel class of noncoding RNA characterized by a ring formation through covalent bonds, play a critical role in various cardiovascular diseases. However, few studies have been conducted to investigate the role and mechanism of circRNA in myocardial lipotoxicity. Here, we found that circ_005077, formed by exon 2–4 of Crmp1, was significantly upregulated in the myocardium of an HFD-fed rat. Furthermore, we identified circ_005077 as a novel ferroptosis-related regulator that plays a role in palmitic acid (PA) and HFD-induced myocardial lipotoxicity in vitro and in vivo. Mechanically, circ_005077 interacted with Cyclophilin A (CyPA) and inhibited its degradation via the ubiquitination proteasome system (UBS), thus promoting the interaction between CyPA and p47phox to enhance the activity of nicotinamide adenine dinucleotide phosphate (NADPH) oxidase responsible for ROS generation, subsequently inducing ferroptosis. Therefore, our results provide new insights into the mechanisms of myocardial lipotoxicity, potentially leading to the identification of a novel therapeutic target for the treatment of myocardial lipotoxicity in the future.

**Supplementary Information:**

The online version contains supplementary material available at 10.1186/s12933-024-02204-3.

## Introduction

The long-term high-fat diet (HFD) causes sharply increased levels of triglycerides (TG) and their hydrolysates, namely, free fatty acids (FFA), in the blood [1]. When the ability of the tissue to oxidize FFA and the storage capacity of adipose tissue is exceeded, triglycerides and FFA ectopic deposits are formed in nonadipose tissue, causing chronic damage to tissue cells and ultimately resulting in dysfunction of target organs, which is called lipotoxicity [2, 3]. The heart is a common target organ for lipotoxicity, which is characterized pathologically by myocardial hypertrophy, fibrosis, and remodeling [4] and clinically by cardiac dysfunction and heart failure in patients with obesity and diabetes [5, 6]. The incidence of heart failure in diabetes and obesity patients is significantly increased, even if adjustment for coronary artery disease, hypertension and significant valvular disease, which proves that diabetes cardiomyopathy or obesity cardiomyopathy, defined as either systolic or diastolic left ventricular dysfunction in otherwise healthy diabetic or obesity persons, objectively exists [5, 7]. Clinical statistics has showed that the prevalence of diabetic cardiomyopathy in population may be approximately 1.1∼1.6% [8, 9]. In addition, the prevalence of diabetic cardiomyopathy in diabetic patients is 16.9% and the prevalence of diastolic dysfunction in diabetic patients is 54% [8]. With an increasing number of patients with obesity and diabetes worldwide, researchers are increasing their efforts on the mechanism for myocardial lipotoxicity. Myocardial lipotoxicity is a complex process involving several factors participated in regulation at multiple levels [4]. The elevated TG and FFA can lead to mitochondrial dysfunction [10] and endoplasmic reticulum (ER) stress [11] at the subcellular level. Meanwhile, many intracellular signal transduction pathways, such as AMP-dependent kinase (AMPK) [12], peroxisome proliferators-activated receptor (PPAR) [13] and NF-κB signal pathways [14], are also activated. In consequence, the phenotypes of inflammation, apoptosis and necrosis occur in myocardial cells [3], thereby resulting in abnormal alteration in the structure of heart and cardiac dysfunction. However, the effects of these regulatory factors are not independent of each other, but rather involve extensive interactions. Thus, the mechanism of myocardial lipotoxicity is far from fully understood.

A novel area that has recently illuminated our understanding of the relations between obesity, diabetes, and lipotoxicity are noncoding RNA, which can regulate gene expression at the post-transcriptional level [15]. For example, in our previous study [16], we found that microRNAs (miRNAs), a short noncoding RNA about 21∼25 nt, are dysregulated in the myocardium of an HFD-induced obesity rat. Furthermore, miR-141 and miR-144 are found to regulate palmitic acid (PA), a kind of saturated fatty acid, induced myocardial hypertrophy and fibrosis in vitro, suggesting that miRNAs play a key role in myocardial lipotoxicity [16]. Moreover, we also have demonstrated that some long noncoding RNAs, such as GAS5 [17], MALAT1 [18] and OIP5-AS1 [19], participates in the regulation of myocardial lipotoxicity. Circular RNAs (circRNA) is another class of noncoding RNA, characterized by a ring formation through covalent bonds and lack of a 5′ terminal cap and a 3′ terminal poly (A) tail [20]. Although the biological functions of circRNAs have not been well understood, our knowledge on its function includes circRNAs can at least regulate gene transcription [22], interact with proteins [21], act as miRNA sponges [22], and translate proteins or peptides [23]. Interestingly, circRNAs play a critical role in various cardiovascular diseases, including acute myocardial infarct [24], dilated cardiomyopathy [25], heart failure [26], and overexpression or downregulation of circRNAs can decrease cardiomyocyte death [27], attenuate myocardial hypertrophy [28], and fibrosis [29], which implies that circRNA is a novel therapeutic target for cardiovascular disease. However, few studies have been conducted to investigate the role and mechanism of circRNA in myocardial lipotoxicity.

Ferroptosis, a new programmed cell death pattern different from apoptosis and autophagy, is an iron-dependent death procedure characterized by an excessive accumulation of lipid peroxides and reactive oxygen species (ROS) [30]. Current knowledge about the main mechanism of ferroptosis is that highly expressed unsaturated fatty acids on the cell membrane are catalyzed by the action of divalent iron or ester oxygenase, leading to lipid peroxidation and ROS accumulation, which subsequently induces cell death [31]. In addition, ferroptosis is also associated with a marked decrease in glutathione peroxidase 4 (GPX4) [32], a core enzyme that regulates the antioxidant system (glutathione system). Mitochondria are the main source of ROS, and the mitochondrial dysfunction in the condition of lipotoxicity caused by diabetes and obesity will produce excessive ROS [10], which implies that myocardial lipotoxicity is closely related to ferroptosis. Mounting evidence has shown that ferroptosis can contribute to cardiac dysfunction and remodeling caused by obesity and diabetes [33, 34]. More importantly, myocardial ferroptosis may be strongly induced when exposed long-term to HFD or FFA [35, 36]. However, the roles of circRNAs in lipotoxicity-induced myocardial ferroptosis are still unclear and need further investigation.

In this paper, circ_005077, formed by exon 2–4 of Crmp1, was significantly upregulated in the myocardium of an HFD-fed rat. Furthermore, we identified circ_005077 as a novel ferroptosis-related regulator that plays a role in PA and HFD-induced myocardial lipotoxicity in vitro and in vivo. Mechanically, circ_005077 interacts with Cyclophilin A (CyPA) and inhibits its degradation via the ubiquitination proteasome system (UBS), thus promoting the interaction between CyPA and p47phox to enhance nicotinamide adenine dinucleotide phosphate (NADPH) oxidase activity and ROS content, subsequently inducing ferroptosis. Therefore, our results provide new insights into the mechanisms of myocardial lipotoxicity.

## Materials and methods

### Animals

Healthy male Wistar rats weighing 200 ± 20 g were purchased from SiPeiFu Biotechnology Co., Ltd (Beijing, China), and fed a normal chow diet (NCD) or HFD (60% ratio of fat to energy supply) (Keao Xieli Feed Co., Ltd, Beijing, China) for 20 consecutive weeks. Each rat was housed in settings of 22 − 24 °C, 12 h of light and darkness, and unrestricted access to clean water. The body weight of the rats was recorded once every 4 weeks. All experimental protocols were approved by the Institutional Animal Care and Use Committee of the China Medical University (CMU2021492) and followed the guidelines provided by the National Institutes of Health (NIH, USA).

### Blood biochemistry analysis

After 20 weeks of feeding, blood samples were collected from the rat’s tail vein after a fast of 6 h. The fasting and OGTT Glucose Tolerance Test glucose levels were detected using a blood glucose meter and glucose test strips (OneTouch Ultra Easy, China). Serum insulin content was measured using the competitive rat insulin enzyme-linked immunosorbent assay (ELISA) Kit (EK3220-96, MULTI SCIENCES, China) following the manufacturer’s instructions. The triglyceride (TG), cholesterol (CHO) and low-density lipoprotein (LDL-C) indexes were analyzed using an automatic biochemical analyzer (Chemray800, Rayto, China).

### Echocardiography

In the 20th week, transthoracic echocardiography was performed using a Vevo 3100LT system with an MX250 probe (center frequency 30 MHz). Briefly, isoflurane was used to anesthetize rats (5% for inducing and 2% for maintaining anesthesia). The rat’s paws were taped to a conductive paste-coated electrode to maintain the correct electrocardiogram (ECG), body temperature, and respiratory rate. The chest wall was exposed after depilation. Images were recorded along the short axis in the middle part of the left ventricle and stored offline for subsequent analysis (VevoLAB3.2.6). The cardiac function parameters obtained are listed in Table S1A.

### Histological analysis

Myocardial tissues were fixed in 4% paraformaldehyde overnight, then they were prepared into 6-µm-thick paraffin sections. To assess the histological change of the myofibers, the sections were stained with hematoxylin and eosin. Special staining (Masson and Sirius red staining) was performed to determine the collagen deposition.

### Circ RNA microarray

Total RNA was extracted from each myocardial sample using TRIzol Reagent (15596-026, Invitrogen, USA) and quantified using NanoDrop ND-1000 (Thermo Fisher, Waltham, MA, USA). For sample preparation and microarray hybridization, the standard Arraystar techniques were used. In short, RNase R (Epicenter, Inc.) was used to digest total RNAs, separate linear RNAs, and enrich circular RNAs. The enriched circular RNAs were then amplified and converted into fluorescent cRNA utilizing a random priming method (Arraystar Super RNA Labeling Kit; Arraystar). The Arraystar Rat circRNA Array (8 × 15 K, Arraystar) was hybridized with the tagged cRNAs. After the slides were washed, the Agilent Scanner G2505C was used to scan the arrays.

Meanwhile, the Agilent Feature Extraction (version 11.0.1.1) software was employed to examine the images obtained in the array. Quantile normalization and subsequent data processing were performed using the R software limma package. Meanwhile, volcano plot filtering was used to identify differentially expressed circRNAs between two statistically significant groups. With fold-change filtering, circRNAs that were differentially expressed between two samples were identified. Hierarchical clustering analysis was performed to display the unique circRNA expression pattern across samples.

### Quantitative real-time polymerase chain reaction

To detect the mRNA level, an aliquot of 800 ng of total RNA was reverse transcribed using the PrimeScriptTM RT reagent Kit with gDNA Eraser (RR047A, TaKaRa, Japan). The cDNA sample was subjected to quantitative real-time polymerase chain reaction (qRT-PCR) using TB Green® Premix Ex Taq TM II(Tli RHaseH Plus)(RR820A, TaKaRa, Japan) and the QuantStudio TM II Real-Time PCR Instrument (A40425,Thermo Fisher Scientific, USA). Gene expression levels were normalized using β-actin. To measure miRNA expression, miRNA was converted into cDNA using the Mir-XTM miRNA First-Stand Synthesis Kit (638,313, TaKaRa, Japan), followed by amplification by qRT-PCR using TB Green® Premix Ex Taq TM II. U6 was used as an intern control for normalization. Moreover, gene expression was analyzed using the 2^−ΔΔCt^ method. The primer sequences used was listed in Table S1E.

### RNA in situ hybridization (ISH)

To detect the expression pattern of circ_005077 in myocardial tissue, RNA ISH using an ISH kit (Boster Biological, CA, USA) was performed. Appropriate amounts of pepsin was added to paraffin sections and digested at 37 °C for 15 min to expose the nucleic acid fragment. The digoxigenin-labeled circ_005077 probe(5‘-CTGTTGTGGTGCCGGTCTCTCTTTGTCCTGCTCTTGCTCC-3’) was hybridized with the tissue after prehybridization. The biotinylated rat antidigoxigenin, the streptavidin–biotin complex (sABC), and the biotinylated peroxidase were then added. Finally, the color develops with DAB for 3 min. Slices were blocked and observed under a microscope (Aperio Versa 8, Leica, Germany).

### Cell culture and PA treatment

The adult rat cardiomyocyte line (H9c2) obtained from the Chinese Academy of Sciences (Shanghai, China) was cultured in low glucose medium containing 10% fetal bovine serum (FBS), 100 units/ml penicillin, and 100 µg/ml streptomycin in a 37 °C, 5% CO_2_ incubator.

Primary neonatal rat ventricular cardiomyocytes (NRVCs) were isolated from 1- to 3-day-old Wistar neonatal rats as previously described [37]. Primary NRVCs were seeded in a 6-well plate with DMEM/F12 medium (11,320,033, Gibco, USA) containing 10% FBS, 100 units/ml penicillin, and 100 µg/ml streptomycin then preincubated for 1.5 h to remove cardiac fibroblasts. Finally, purified NRVCs were cultured in a CO_2_ incubator at 37 °C for 72 h.

In order to induce in vitro myocardial lipotoxicity, as described in a previous article [8]. H9c2 cells were treated for 24 h with 250 µM PA (Sigma-Aldrich, St. Louis, MO), whereas NRVCs were treated for 48 h with 150 µM PA.

### RNA fluorescence in situ hybridization(FISH)

RNA-FISH was performed according to the manufacturer’s instructions using the FISH detection kit (G3017, Servicebio, Wuhan, China). The circ_005077 specific probe (5′AGGAGCCGGTCACTCTTTGTCCTGCACTAG3′) was designed and synthesized by Servicebio. After prehybridization treatment, cells cultured on slides were incubated with probe hybrid solution (1µM) in a wet box at 37 °C overnight, After washing with sodium citrate saline buffer, DAPI was added to re-dye the nucleus. Cells were observed and analyzed under a confocal microscope (AXR, Nikon, Japan).

### RNA stability testing

H9c2 cells were treated with 5 µg/ml actinomycin D (HY-17,559, MCE, USA) when they reached 70–80% confluence in 6-well plates and collected at different time points. Total RNA was extracted from collected cells and the levels of circ_005077 and the corresponding linear RNA (crmp1 mRNA) were analyzed by qRT-PCR.

For the RNase R treatment, 5 µg total RNA was incubated for 15 min at 37 °C with or without 3 U/µg RNase R (RNR16404, Lucigen, USA). qRT-PCR was used to detect circ_005077 and crmp1 mRNA levels after RNase R treatment.

### Transfection of cells and establishment of stable transformation cell lines

Hanbio Tech (Shanghai, China) designed and synthesized the plasmids (pcDNA3.1-Ppia, pcDNA3.1-Ncf1, and their control vector pcDNA3.1). Meanwhile, small interfering RNAs (siRNAs), including siRNA-circ_007077, siRNA-Ppia, siRNA-Ncf1, and negative control were designed and synthesized by RiboBio (Guangzhou, China). According to the manufacturer’s protocols, the plasmids and siRNAs were transfected into cells using the Lipofectamine 3000 Transfection Kit (Invitrogen, Carlsbad, CA).

Lentivirus carrying the circ_005077 sequence or short hairpin RNA (shRNA) targeting rno-circ_005077 and their corresponding control vectors designed and synthesized by Hanbio Tech were used to infect H9c2 cells. Forty-eight hours after infection, cells were subjected to puromycin treatment for two weeks to construct cell lines that stably overexpress or silence circ_005077.

### RNA sequencing

Total RNA was extracted and quantified in the same manner as mentioned earlier. The RNA was tested for integrity using a Bioanalyzer 2100 (Agilent, CA, USA) and confirmed using agarose electrophoresis. PolyA (polyadenylate) RNA was specifically captured using Dynabeads Oligo (dT)25-61005, (Thermo Fisher, CA, USA) through two rounds of purification. The captured mRNA was fragmented into small pieces at 94 °C for 5–7 min using the magnesium RNA fragmentation module (E6150, NEB, USA). SuperScript™ II synthesized cDNA reverse transcriptase (1,896,649, Invitrogen, USA), then *E. coli* DNA polymerase I (m0209, NEB, USA) and RNase H (m0297, NEB, USA) were used for double-stranded synthesis to synthesize U-labeled second-stranded DNAs, dUTP solution (R0133, Thermo Fisher, USA) was added to make the ends of double-stranded DNA flat. An A-base was added to each end to connect with the terminal joint with a T-base; then, the AMPureXP beads were used to screen and purify their fragment size. The U-labeled second-strand DNAs were digested with the UDG enzyme (m0280, NEB, USA), then predenatured by PCR, at 95 ° for 3 min, denatured at 98 ° for 15 s with eight cycles, annealed at 60 ° for 15 s, extended at 72 ° for 30 s, and finally extended at 72 ° for 5 min. The average insert size for the final cDNA library was 300 ± 50 bp. Ultimately, the vendor-recommended methodology was followed while performing the paired-end sequencing (PE150) on an Illumina NovaseqTM 6000 (LC-Bio Technology Co., Ltd., Hangzhou, China).

Based on the analysis of significant differences between samples, genes with a difference FC > 2 times or a difference FC < 0.5 times and a p-value of < 0.05 were defined as differential genes, and then Gene Ontology (GO) and Kyoto Encyclopedia of Genes and Genomes (KEGG) enrichment analysis was performed.

### Chromatin isolation by RNA purification (ChIRP)

The proteins binding to circRNA were detected using the ChIRP method as previously described [38]. The biotin-labeled antisense probes were designed at the back-spliced site of circ_005077 and synthesized by RiboBio. A total of 2 × 10^7^ cells were collected, cross-linked with 3% formaldehyde solution for 30 min at room temperature, and then cell lysate was prepared using lysis buffer. The biotin-labeled probes were combined with magnetic beads for 30 min, followed by mixing with the sample for hybridization overnight at 37 °C. The magnetic beads were washed with 1 ml of wash buffer at 37 °C five times, collected using a magnetic frame, and mixed with protein elution buffer and dithiothreitol at 37 °C for 2 h. Magnetic beads were re-collected, and the supernatant (protein sample) was transferred to a new centrifuge tube for chromatography-mass spectrometry/mass spectrometry (LC-MS/MS) or Western blotting detection.

### LC-MS/MS

The nano-UPLC liquid phase system EASY-nLC1200 was used to divide each sample into 5 µl polypeptides, and the polypeptides were identified using an online mass spectrometer (Q-Exactive). The enzymatic hydrolysate was separated via nano-UPLC and then analyzed online using a Q-Exactive mass spectrometer (Thermo Finnigan). MaxQuant (version 2.0.1.0) searched and quantitatively analyzed the LC-MS/MS raw data. The protein database was uniprot-proteome-rat-2021.2. FASTA. To enrich U1snRNA and identify the binding protein, a specific probe of U1snRNA was used as the positive control group (PC). Nonspecific antisense was used in the negative control group. The proteins precipitated by the circ_005077 probes were quantified based on the fold-change of normalized spectral counts relative to the negative control. The results of the ChIRP-MS assay are listed in Table S5B.

### RNA immunoprecipitation (RIP) and immunoprecipitation (IP)

Magna RIP RNA-Binding Protein Immunoprecipitation Kit (17–700, Millipore, USA) was used for RIP experiments in accordance with previously mentioned manufacturer’s instructions [39]. Cell lysates were incubated with 5 µg of CyPA primary antibodies (sc-134,310, Santa Cruz Biotechnology, USA). Mouse immunoglobulin (IgG) was used as a control. Western blot and qRT-PCR were used to detect the final product.

IP experiments were used with the IP Kit with Protein A + G Magnetic Beads (P2179S, Beyotime Biotechnology, China) and the Flag-Tag Protein IP Assay Kit with Magnetic Beads (P2181S, Beyotime Biotechnology, China). In Brief, the pretreated magnetic beads and the diluted CyPA (1 µg per 100 µg of total protein) of tris-buffered saline were flipped and incubated on a flip mixer for 1 h at room temperature. The above step was omitted in flag-tag magnetic beads. The magnetic beads were then washed three times and incubated overnight with protein sample lysis buffer at 4 °C on a rotary mixer. The next day, the magnetic beads were eluted with SDS-PAGE sampling buffer, followed by Western blot analysis.

### Transmission electron microscopy

In 2.5% glutaraldehyde (G1102, Sevicebio, China), 1mm^3^ fresh rat heart tissue or 1 × 10^7^ H9c2 cells were fixed overnight at 4 °C. After being rinsed, post-fixed with 1% osmium tetroxide, and gradient dehydration with ethanol, the samples were inserted into the mold in a 37 °C oven overnight. Following entrapment (90529-77-4, SPI, China), ultrathin sections were prepared and stained twice with uranyl acetate and lead citrate. Observation and image analysis under a transmission electron microscope (TEM) (HT7700, Hitachi, Japan).

### Glutathione (GSH), malondialdehyde (MDA), lactate dehydrogenase (LDH), and coenzyme II NADP(H) content ass

The determination of the content of GSH (BC1175, Solarbio, China), MDA (S0131, Beyotime, China), LDH (BC0685, Solarbio, China) content and the NADPH/NADP^+^ ratio (BC1105, Solarbio, China) was carried out according to the manufacturer’s instructions.

### Detection of ROS and Liperfluo (LPO) content

Cytoplasmic ROS and mitochondrial ROS were detected by DCFH-DA (S0033S, Beyotime Biotechnology, China) and MitoSOX Deep Red (MT14, Dojindo Laboratories, Japan) following the manufacturer’s instructions, respectively. Briefly, DCFH-DA and MitoSOX were diluted to 1: 1000 dilution in serum-free medium to a final concentration of 10 μm. Cell culture fluids were removed and incubated with the above working solution for 30 min at 37 °C in the dark. Fluorescent signals were quantified by Image J (version 1.53e, National Institutes of Health, USA).

For the detection of cellular LPO, cells were incubated with 5 mm LPO (L248, Dojindo Laboratories, Japan) for 30 min at 37 °C in the dark. Fluorescence images were captured by a confocal microscope and analyzed using Image J.

### Measurement of Fe2 + content

Cells were incubated in the dark for 30 min at 37 °C with 1 µM FerroOrange working solution (F374, Dojindo Laboratories, Japan) to measure cellular Fe2 + content. A confocal microscope was used to capture the fluorescence images, which were then analyzed using Image J.

### Protein stability testing

To test whether CyPA stability was affected by circ-005077, H9c2 cells that stably overexpress or silencing rno-circ_005077 were treated with 10 µM proteasome inhibitor MG-132 (HY-13,259, MCE, USA) or 30 µM protein synthesis inhibitor cycloheximide (CHX) (HY-12,320, MCE, USA) according to the previous report [40]. After treatment, the proteins were isolated and the expression of CyPA was detected by Western blotting.

#### Measurement of NADPH oxidase activity

The levels of NADPH oxidase (NOX) activity levels in rats were measured via ELISA using a NADPH oxidase detection kit (Fusheng Biotech Ltd., Shanghai, China) following manufacturer’s instructions. The absorbance (optical density) was measured via a microplate reader (UV-visible Spectrometer Uv300, UK) at a wavelength of 450 nm, and the activity of NOX in the sample was calculated by standard curve.

### Western blotting

Cells were harvested and lysed with RIPA buffer supplemented with protease and phosphatase inhibitors on ice for 30 min. Protein concentration was quantified using the BCA Protein Assay Kit (P0012, Beyotime Biotechnology, China) Equal amounts of protein were electrophoretically separated on 7.5% or 12.5% polyacrylamide gels before being transferred to polyvinylidene fluoride membranes. The membranes were incubated with primary antibodies after blocking with Protein Free Rapid Blocking Buffer (PS108P, EpiZyme Biotechnology, China) for 45 min at room temperature, including rabbit polyclonal to CyPA (1:2000, ab41684, abcam), mouse monoclonal to CyPA (1:1000, sc-134,310, Santa Cruz Biotechnology), rabbit monoclonal to NCF1/p47-phox (1:1000, ab308256, abcam), rabbit monoclonal to FTH1 (1:1000, ab183781, abcam), rabbit monoclonal to GPX4(1:5000, ab125066, abcam), rabbit monoclonal to FACL4(1:10000, ab155282, abcam), rabbit monoclonal to COX2 (1:5000, ab179800, abcam), mouse monoclonal to transferrin receptor (1:5000, ab269513, abcam), rabbit monoclonal to PDIA6 (1:10000, ab154820, abcam), mouse monoclonal to P4HB (1:1000, ab2792, abcam), rabbit polyclonal to ERp57/ERp60 (1:2000, 15967-1-AP, proteintech), mouse to DYKDDDDK-Tag(1:5000, M20008, abmart), mouse to HA-Tag (1:5000, M20003, abmart), rabbit polyclonal to ubiquitin(1:1000, 10201-2-AP, proteintech), or rabbit recombinant antibody to Beta Actin(1:10000, 81115-1-RR, proteintech) at 4 °C overnight. Subsequently, the membranes were washed three times, followed by incubation with HRP-conjugated affinipure goat anti-rabbit IgG (H + L) (1:10000, SA00001-2, proteintech) or HRP-conjugated affinipure goat anti-mouse IgG (H + L) (1:10000, SA00001-2, proteintech) secondary antibodies for 1 h at room temperature. ECL chemiluminescence was captured using the automatic chemiluminescence image analysis system (5200, Tanon, China).

### Immunohistochemistry (IHC)

The paraffin sections were deparaffinized in xylene and rehydrated in alcohol. After antigen recovery, the slices were treated with hydrogen peroxide (H_2_O_2_) and goat serum, followed by incubation with anti-FTH1(1:200, ab183781), anti-GPX4 (1:200, ab125066), anti-ACSL4(1:200, ab155282), anti-TFRC (1:200, ab269513) and anti-COX2 (1:200, ab179800) at 4 °C overnight. After being washed three times, the slices were further incubated with secondary antibodies at 37 °C for 1 h. Following hematoxylin staining and differentiation with 1% hydrochloric acid alcohol, these slices were dehydrated in alcohol, xylene transparent, and photographed under a microscope (Aperio Versa 8, Leica, Germany).

### Statistical analysis

Data were presented as mean ± standard deviation (SD) and statistically analyzed with SPSS version 17.0 software (SPSS, Inc., Chicago, IL, USA). The Student’s t-test was used to examine the variations between the two groups. A one-way analysis of variance was first used to examine differences between more than two groups. Multiple comparison analysis was conducted using Fisher’s least significant difference test when appropriate and suitable. A statistically significant difference was defined as *P* < 0.05.

## Results

### Expression and characterization of circ_005077 in the HFD rat heart

**Fig. 1:**
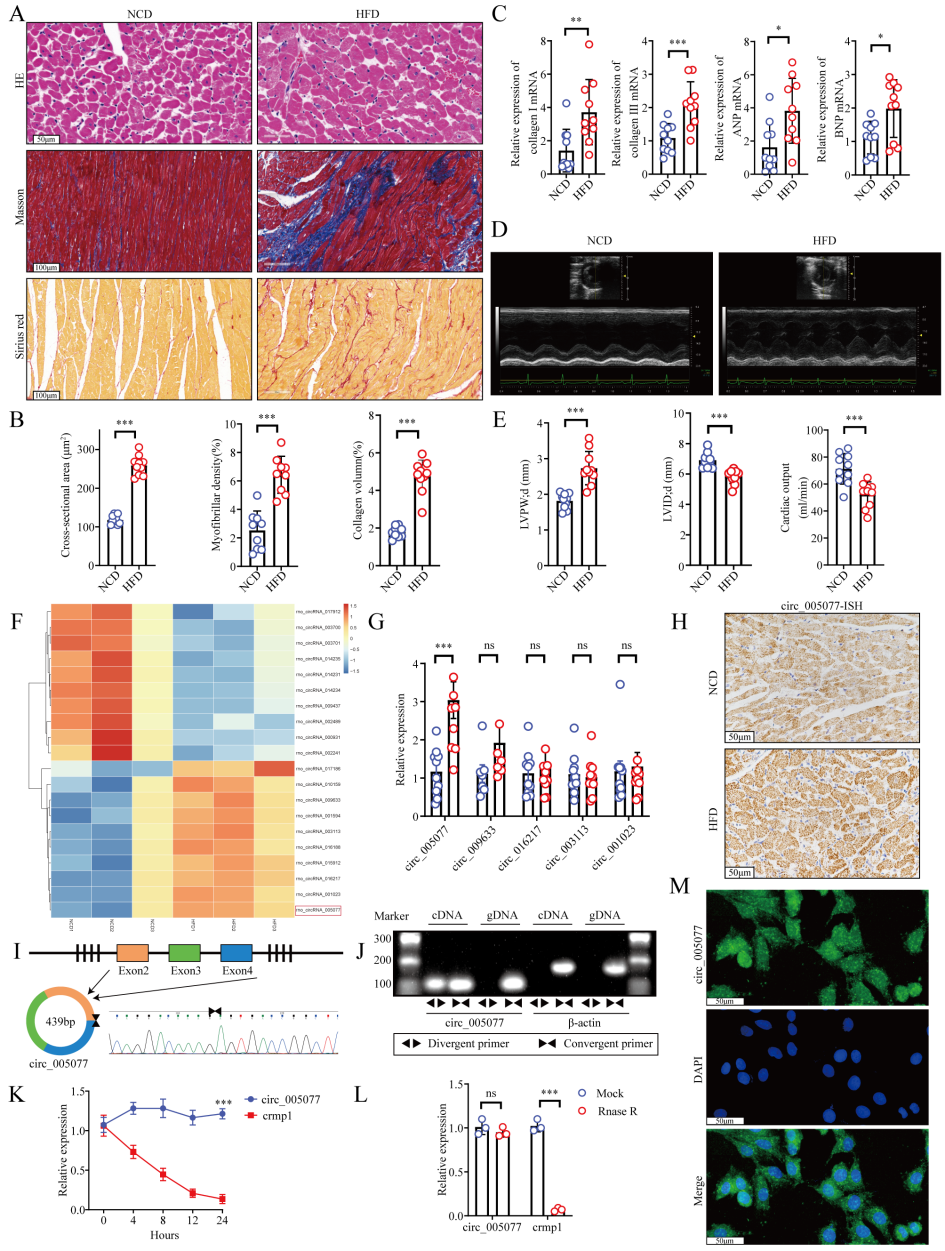
Identification and characteristics of circ_005077 in H9c2 cells and myocardial tissues. Rats were fed a normal chow diet (NCD) or high-fat diet (HFD) for 20 consecutive weeks and heart tissues were harvested for detection. (**A**, **B**) Representative image of hematoxylin and eosin staining representative image (400× magnification) with the cross-sectional area of the cardiomyocyte evaluated; Masson staining (200× magnification) with the myofibrillar density evaluated; Sirius Red staining (200× magnification) with the collagen volume evaluated. (**C**) Analysis of relative expression of hypertrophy and fibrosis indicators (ANP, BNP, Collagen I, and Collagen III) in the myocardium identified by qRT-PCR. (**D**, **E**) Typical images taken in M mode. Measurement of the thickness of the left ventricular posterior wall at the end-diastole (LVPW; d) left ventricular end-diastolic chamber diameters (LVID; d), and cardiac output (CO). (**F**) Hierarchical clustering heatmap showing differences in the circRNAs of greatest expression between NCD and HFD myocardium with the top 10 upregulated or downregulated genes selected (*n* = 3, *P* < 0.05). In a heat map, red indicates upregulation. Blue denotes downregulation. (**G**) qRT-PCR validation of microarray-based expression of the top five upregulated circRNAs. Scatter plots displayed the results of ten independent experiments conducted for each group. (**H**) In situ hybridization showed the expression of circ_005077in NCD and HFD myocardial tissue Sect. (400× magnification). (**I**) Exons 2–4 of the *crmp1* gene were used to generate circ_005077 in the schematic diagram (top). Circ_005077 was identified by Sanger sequencing in H9c2 cells. The black arrow marked the back-splicing site conjunction. (**J**) Circ_005077 and β-actin were detected from the cDNA or genomic DNA (gDNA) of the H9c2 cell using divergent and convergent primers by agarose gel electrophoresis experiments. (**K**) Circ_005077 and Crmp1 abundance were determined by qRT-PCR in H9c2 cells treated with actinomycin D at the specified time points (*n* = 4). (**L**) qRT-PCR for circ_005077 and crmp1 abundance in H9c2 cells treated with RNase R versus mock cells (*n* = 3). (**M**) RNA-FISH test demonstrated the localization of circ_005077 in the cytoplasm and nucleus regions of H9c2 cells using a junction-specific antisense probe (green, 400× magnification) and nuclear labeling with DAPI (blue, 400× magnification). Data were presented as mean ± SD. *P < 0.05, ** *P* < 0.01, *** *P* < 0.001, NS, no significance

After 20 weeks of feeding, body weight, serum glucose, TG, CHO, and LDL-C levels in HFD-fed rats increased significantly compared with NCD-fed rats (Fig S1). Simultaneously, HFD-fed rats manifested hypertrophy and fibrosis of the myocardium (Fig. 1A and B), upregulated hypertrophy-and fibrosis-related genes (Collagen I, Collagen III, ANP, and BNP) (Fig. 1C), and impaired cardiac function (Fig. 1D and E). Analysis of the circRNA microarray discovered 21 significantly upregulated circRNAs and 186 downregulated circRNAs (Table S1C, D and Fig. 1F). Furthermore, we selected the top five upregulated circRNAs for validation and, finally, found that circ_005077 expression was significantly increased by 3-folds in the HFD rat heart (Fig. 1G). Moreover, the results of tissue in situ hybridization also showed that the expression of circ_005077 in the HFD rat myocardium was much higher than in the NCD rat myocardium (Fig. 1H).

Circ_005077 was created by back-splicing Crmp 1 exons 2, 3, and 4 (Fig. 1I). RT-PCR with convergent and divergent primers was used to validate the existence of circ_005077’s head-to-tail splicing. As shown in Fig. 1J, circ_005077 was only detected in cDNA but not genomic DNA (gDNA) with divergent primers, confirming the presence of circ_005077. Cells were treated with RNase R and actinomycin D to further confirm the stability of circ_005077, and it was discovered that circ_005077 was resistant to both actinomycin D and RNase R treatment (Fig. 1K and L), indicating that circ_005077 was more stable than Crmp 1 mRNA. FISH showed that circ_005077 was located in the cardiomyocyte’s nucleus and cytoplasm (Fig. 1M).

### Circ_005077 regulates PA-induced myocardial lipotoxicity in vitro

**Fig. 2:**
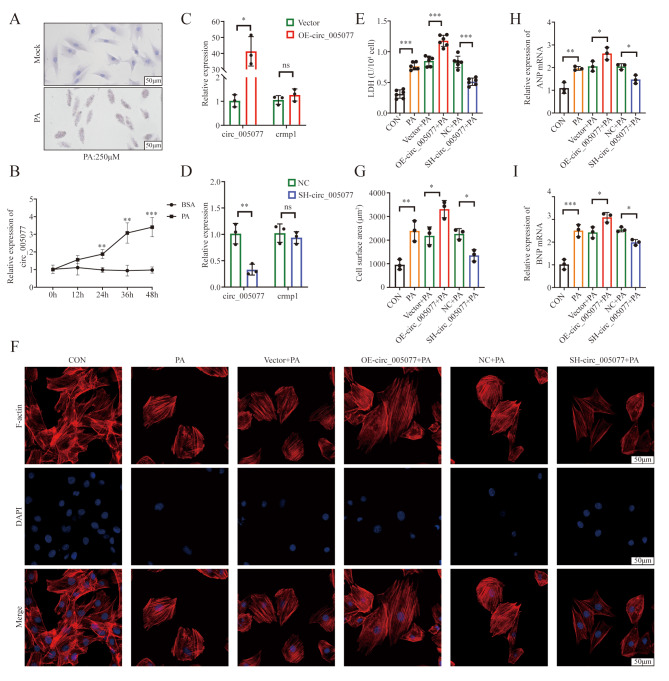
Circ_005077 regulates palmitate (PA)-induced myocardial lipotoxicity in vitro. H9c2 cells or primary neonatal rat ventricular cardiomyocytes (NRVCs) were transfected with OE-circ_005077 mediated by lentivirus (10MOI), vector, SH-circ_005077 (10MOI)), and NC respectively. (C–I) The H9c2 were then given a 24 h treatment with 250 µM PA and the NRVCs were followed by treatment with 150 µM PA for 48 h (E–I). (**A**) Oil-red O staining exhibited lipid deposition in PA-treated H9c2 cells (400× magnification). (**B**) The abundance of circ_005077 in H9c2 cells treated with 250 µM PA at the defined time points was determined by qRT-PCR compared to the treatment of BSA (*n* = 3). (**C**) Circ_005077 overexpression efficacy of OE-circ_005077 compared to vector in H9c2. (**D**) Circ_005077 knocks down the efficacy of SH-circ_005077 compared to NC in H9c2. (**E**) LDH released from H9c2 was measured via colorimetry. (**F** and **G**) Immunofluorescence (IF) of NRVCs for F-actin (red, 600× magnification) and DAPI (blue, 600× magnification). Following IF, cell surface area was measured using morphometry (each dot represents the mean area of a single experiment). (**H** and **I**) Analysis of the relative expression of ANP and BNP mRNA in NRVCs. The results of three to six biological replicates were displayed using scatter plots. The data were presented as mean ± SD. *P < 0.05, **P < 0.01, ***P < 0.001, NS, no significance

Cardiomyocytes were treated with PA to mimic myocardial lipotoxicity *in vitro.* After PA treatment, it was found that the lipid was abundantly deposited in cardiomyocytes (Fig. 2A), and the level of circ_005077 increased significantly 12, 24, 36, and 48 h after treatment (Fig. 2B). To explore the role of circ_005077 in myocardial lipotoxicity, circ_005077, but not Crmp1 was overexpressed and silenced using the lentivirus-carried circ_005077 plasmid and the shRNA sequence (Fig. 2C and D), followed by PA treatment. As shown in Fig. 2E∼I, overexpression of circ_005077 significantly reduced cell viability, increased LDH release, cell surface area, and hypertrophy marker expression (ANP and BNP). Meanwhile, downregulation of circ_005077 clearly increased cell viability and decreased LDH release, cell surface area, and hypertrophy marker expression (ANP and BNP). The above results suggest that circ_005077 could regulate PA-induced myocardial lipotoxicity *in vitro.*

### Circ_005077 acts as a regulator for PA-induced ferroptosis in the cardiomyocyte

**Fig. 3:**
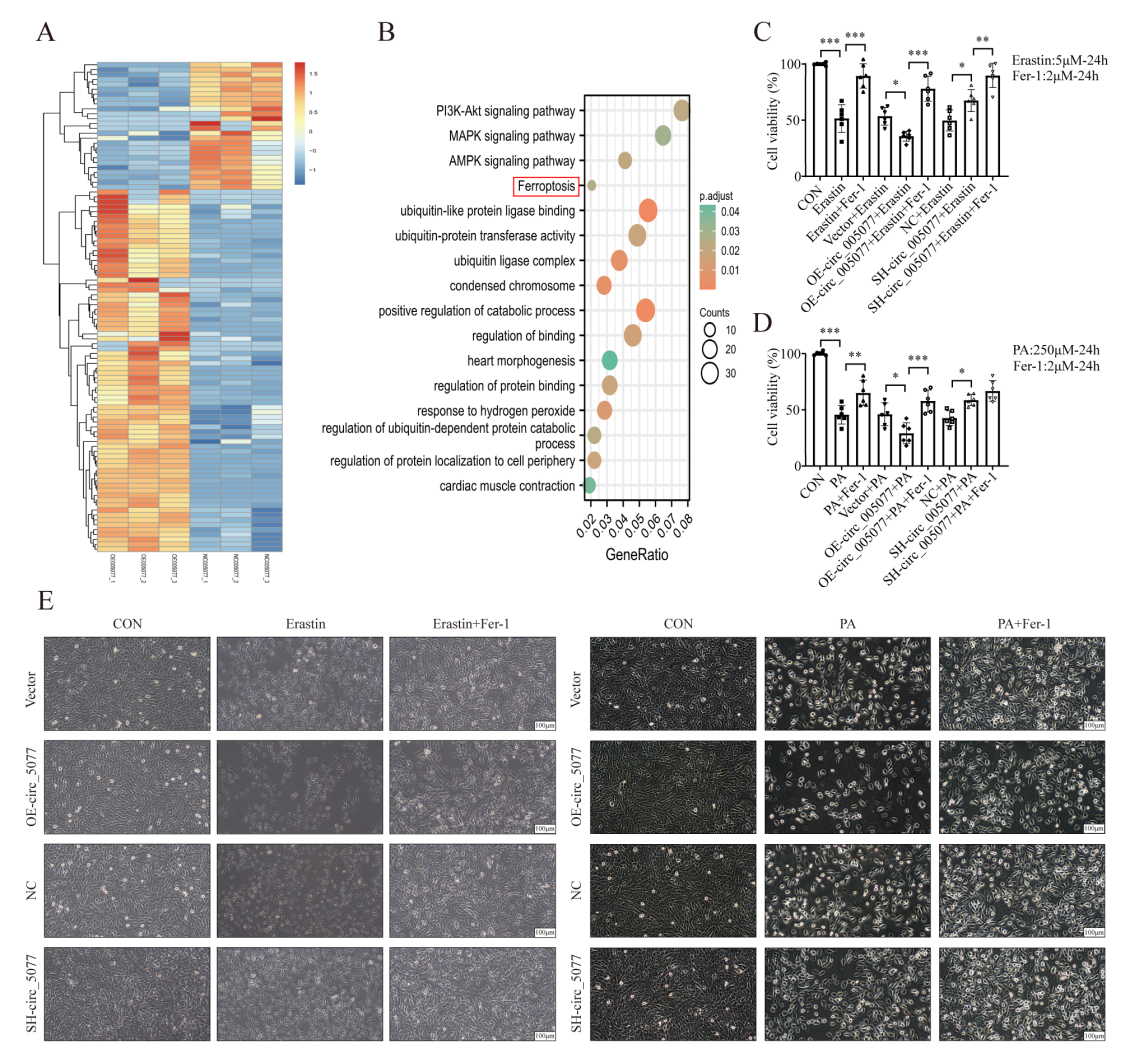
Circ_005077 is involved in the ferroptosis signal pathway. H9c2 cells were transfected with lentivirus-mediated OE-circ_005077 and vector. (**A**) The transcriptomes of the RNA samples were profiled by RNA sequencing (RNA-Seq). Cluster analysis of differential genes (DEGs) between the OE_circ_005077 and vector groups. Heat map taken from the top 100 genes with the lowest p-value. Red indicates high gene expression and blue indicates low gene expression. (**B**) The findings of the KEGG pathways of DEGs, GO biological process enrichment, GO cellular component enrichment, and GO molecular function enrichment. H9c2 cells were treated with erastin (5 µM) or PA (250 µM), with or without of Fer-1(2 µM) for 24 h (C–E). (**C**, **D**) The CCK-8 assay was used to determine cell viability. (**E**) The morphology of the H9c2 was observed using a light microscope. The results of six biological replicates were displayed using scatter plots. Data were presented as mean ± SD. *P < 0.05, **P < 0.01, ***P < 0.001

**Fig. 4:**
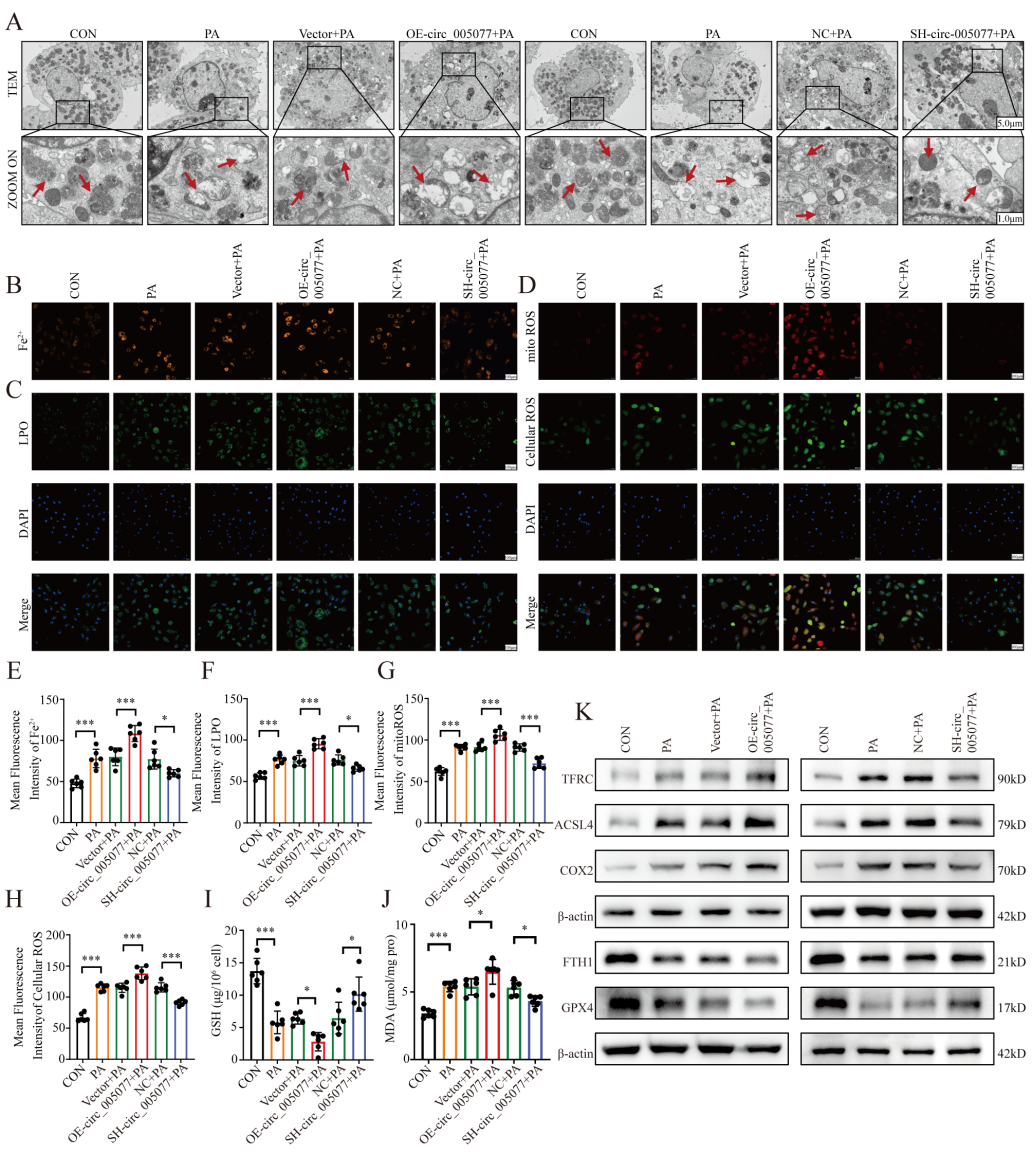
Circ_005077 acts as a regulator for palmitate PA-induced ferroptosis in cardiomyocyte. H9c2 cells were transfected with OE-circ_005077 mediated by lentivirus (10MOI), vector, SH-circ_005077 (10MOI), and NC, respectively (C–I), followed by 250 µM PA treatment for 24 h. (**A**) Representative pictures of H9c2 mitochondria structure under a transmission electron microscope (1,500×, upper row and magnified views at 6,000, lower row). (**B**, **E**) Illustrations and quantitative analysis of ferrous iron (orange, 400× magnification). (**C**, **F**) Illustrations and quantitative analysis of LPO (green, 400× magnification). (**D**,**G**,**H**) Illustrations and quantitative analyses of mitoROS (red, 400× magnification) and cellular ROS (green, 400× magnification). (**I**) Intracellular GSH content. (**J**) Intracellular MDA content. (**K**)Protein levels of TFRC, ACSL4, COX2, FTH1, and GPX4 in treated H9C2 detected via Western blotting. Each point showed the average fluorescence intensity of a single experiment. Data were shown as mean ± SD of at least three independent experiments. *P < 0.05; **P < 0.01; ***P < 0.001

 To explore the downstream pathway influenced by circ_005077, H9c2 cells stably overexpressed with circ_005077 were subjected to RNA sequence (Fig. 3A), followed by GO and KEGG analysis. Among the enriched signaling pathways, the ferroptosis signaling pathway has piqued our interest, as recent research has shown that ferroptosis is involved in the process of HFD-induced myocardial lipotoxicity (Fig. 3B) [35, 36]. Therefore, we first determined circ_005077’s ability to regulate myocardial ferroptosis. Cardiomyocytes were treated with erastin to induce myocardial ferroptosis. Overexpression of circ_005077 exacerbated erastin-induced cell death, whereas downregulation of circ_005077 prevented erastin-induced cell death (Fig. 3C and E). As PA can induce myocardial lipotoxicity and trigger ferroptosis in myocardial cells [41], we further investigated the role of circ_005077 on PA-induced ferroptosis. As indicated in Fig. 3D and E, cell viability decreased by PA treatment was further aggravated in H9c2 cells stably overexpressed with circ_005077, which was rescued by Fer-1 treatment. Moreover, after PA treatment, TEM observation revealed that mitochondria decreased in size with increasing double-layer membrane density, a morphological feature of ferroptosis (Fig. 4A). Meanwhile, PA treatment has been shown to significantly increase the Fe^2+^ content of myocardial cells (Fig. 4B), generation of ROS within cells and mitochondria (Fig. 4D), increase the levels of lipid peroxide (LPO and MDA) levels (Fig. 4C and J), decrease the activity of the antioxidant enzyme GSH (Fig. 4I), increase the expression of TFRC, ACSL4, COX2, and decrease the expression of FTH1and GPX4 (Fig. 4K), which confirms that PA could induce ferroptosis in cardiomyocytes. Furthermore, it was also found that overexpression of circ_005077 could promote the PA-induced ferroptosis-related phenotype in vitro, whereas inhibition of circ_005077 showed opposite effects. Taken together, these results suggest that circ_005077 acts as a regulator for PA-induced ferroptosis in cardiomyocytes.

### Circ_005077 interacts with CyPA and inhibits its degradation

**Fig. 5:**
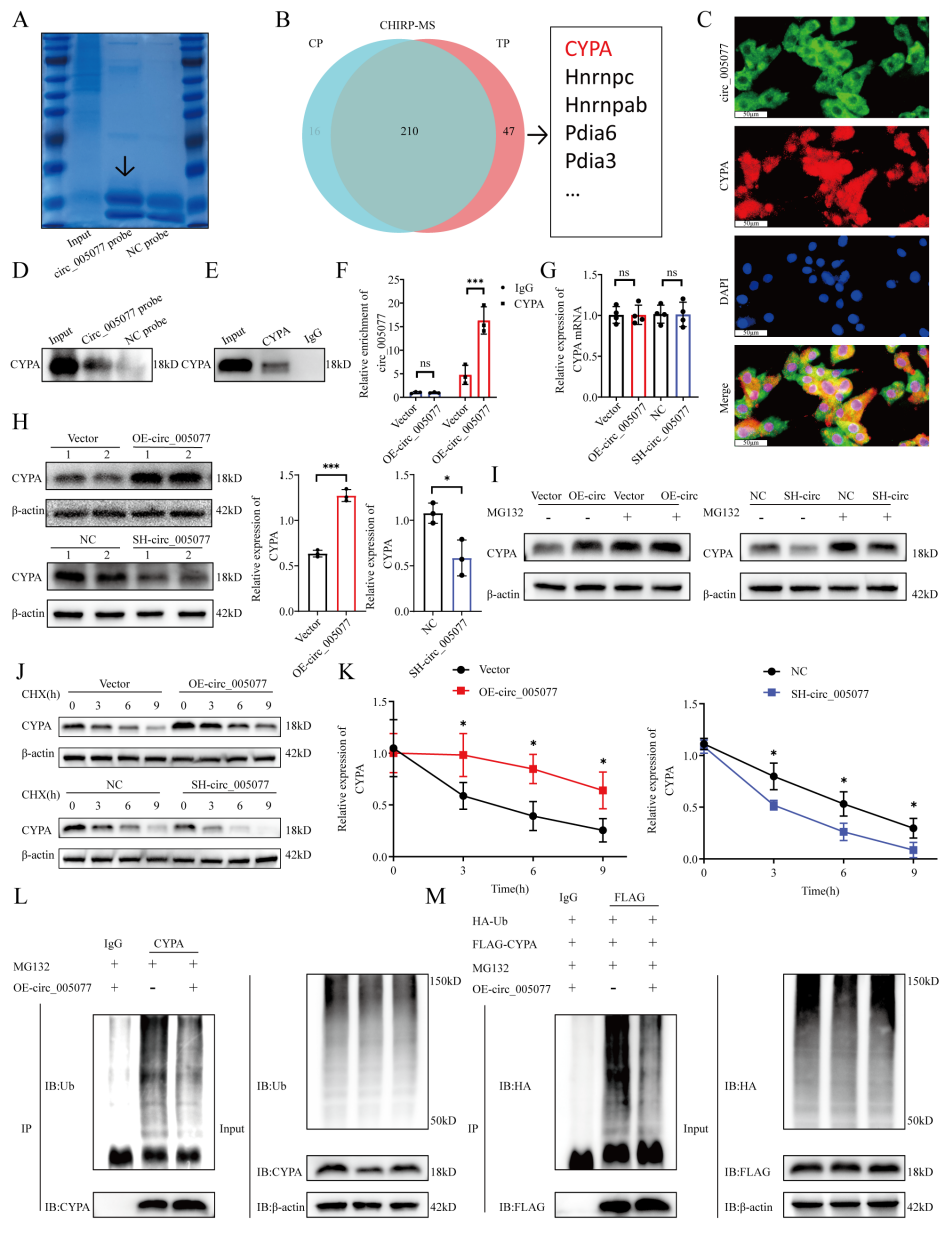
Circ_005077 bound to the CyPA protein and decreased its degradation via the ubiquitin-mediated proteasomal pathway. (**A**) The gel was stained with Coomassie bright blue to show the total proteins loading of ChIRP by circ_005077 probes and NC probes. (**B**)Venn diagram showed the differential proteins of LC-MS analysis. (**C**) H9c2 cells were stained with immunofluorescence to detect the colocalization of the CyPA protein (red, 400× magnification) and circ_005077 (green, 400× magnification). (**D**) The binding of circ_005077 to the CyPA protein was validated by ChIRP assay followed by Western blotting. (**E**) The CyPA protein was pulled down by RIP assay. (**F**) Enrichment of circ_005077 pulled down by the CyPA protein was shown. (**G**) The relative levels of CyPA mRNA in H9c2 cells with inhibition or overexpression of circ_005077 were shown. (**H**) CyPA protein levels were shown in H9c2 cells with inhibition or overexpression of circ_005077. (**I**) The expression of CyPA in H9c2 cells after inhibition or overexpression of circ_005077 and then treated with or without MG132 for 12 h, demonstrated by Western blotting. (**J**) The expression of CyPA was evaluated at different durations of CHX administration with inhibition or overexpression of circ_005077 in H9c2 cells. (**K**) Quantification of CyPA degradation rate by grayscale analysis. (**L**) Ubiquitinated CyPA measured by immunoprecipitation with anti-CyPA antibody or IgG control and immunoblotting with anti-ubiquitin antibody in H9c2 cells after overexpression of circ_005077 or not. (**M**) Flag-CyPA and HA-Ub were co-transfected and expressed in H9c2 cells. Ubiquitinated Flag-CyPA measured by IP with anti-Flag antibody or IgG control and immunoblotting with anti-HA antibody after overexpression of circ_005077 or not. Data were shown as mean ± SD of at least three independent experiments. *P < 0.05; **P < 0.01; ***P < 0.001, NS, no significance

We used MS to identify circ_005077-pulled down proteins to better understand the regulatory mechanism of circ_005077 in PA-induced ferroptosis. The findings revealed 47 potential interacting proteins (Fig. 5A and B, Table S5B). We focused on CyPA, a known ROS regulator [42], through systematic literature review, for further investigation. A combination of FISH and IF staining indicated that circ_005077 and CyPA were co-located in H9c2 cells (Fig. 5C). ChIRP assays confirmed that circ_005077 interacts with CyPA from H9c2 cell extracts (Fig. 5D). Furthermore, RIP assays verified that circ_005077 was enriched with the anti-CyPA antibody compared to those with control IgG (Fig. 5E and F).

To explore the regulation of circ_005077 on CyPA, we further detected the expression of CyPA under upregulation or downregulation of circ_005077. The results showed that overexpression or underexpression of circ_005077 led to an increase or decrease in the expression of the CyPA protein; however, no significant changes were displayed in the expression of CyPA mRNA (Fig. 5G and H). Therefore, we inferred that circ_005077 could regulate the stability of the CyPA protein at the posttranscriptional level. As expected, MG132 treatment was found to abrogate upregulation or downregulation of the CyPA protein by overexpression or underexpression of circ_005077(Fig. 5I). Furthermore, overexpression or underexpression of circ_005077 could lengthen or shorten the half-life of CyPA protein (Fig. 5J and K). The ubiquitination level of CyPA was determined to confirm whether circ_005077 influenced CyPA protein stability via UBS (Fig. 5L and M). As shown, the level of CyPA ubiquitination decreased, relatively, after overexpression of circ_005077 compared to the empty vector group, suggesting that circ_005077 could modulate CyPA ubiquitination.

### Circ_005077 promotes the interaction between CyPA and p47PHOX to enhance NADPH oxidase activity

**Fig. 6:**
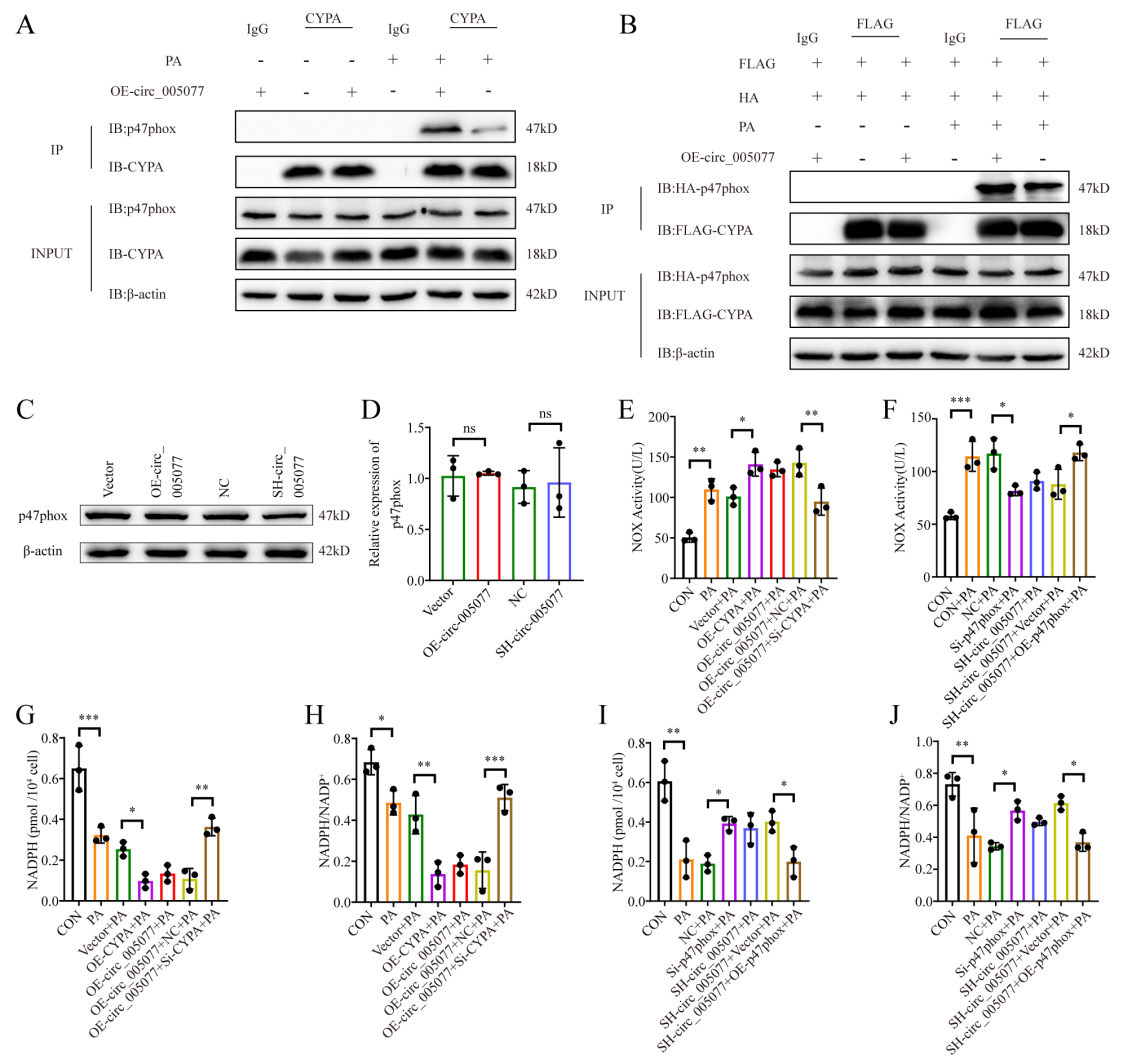
Circ_005077 acted as a scaffold to promote the binding of CyPA and p47phox and activated NADPH oxidase. (**A**) The binding of CYPA to p47phox was determined by Co-IP and Western blotting in H9C2 cells with or without rno_circ_005077 overexpression. (**B**) Flag-CyPA and HA-p47phox were co-transfected and expressed in H9c2 cells with or without overexpression of circ_005077; Co-IP and Western blotting determined the binding of Flag-CyPA to HA-p47phox. (**C**, **D**) Protein levels of p47phox in H9c2 cells with inhibition or overexpression of circ_005077 were shown. (**E**,**F**) The ELISA assays showed the NOX activity of H9C2 cells with overexpression or inhibition of circ_005077, and inhibition of CyPA or overexpression of p47phox can rescue this effect under PA.(**G**,**H**) NADPH and the NADPH/NADP^+^ ratio were measured in H9c2 cells with overexpression of circ_005077, and inhibition of CyPA can reverse this impact under PA.(**I**,**J**) The levels of NADPH and the NADPH/NADP^+^ ratio were measured in H9c2 cells with inhibition of circ_005077, and p47phox overexpression can reverse this impact under PA.Data were shown as mean ± SD of at least three independent experiments. *P < 0.05; **P < 0.01; ***P < 0.001. NS, no significance

According to reports from the literature [42], under the action of angiotensin II, CyPA could affect the activity of NADPH oxidase and regulate ROS generation by interacting with p47PHOX. Therefore, we inferred that circ_005077 might affect the interaction between CyPA and p47PHOX, thus regulating NADPH oxidase activity. As expected, the interaction between CyPA and p47PHOX was remarkably enhanced under the condition of PA treatment, and their interaction was much stronger in the cell overexpressing circ_005077 than in the cell transfected with the empty vector (Fig. 6A and B). However, circ_005077 overexpression or knockdown had little impact on p47PHOX expression (Fig. 6C and D). Furthermore, NADPH oxidase activity increased or decreased in circ_005077 cell upregulation or downregulation compared to cells transfected with empty vectors or NC under PA treatment (Fig. 6E and F), meanwhile, the level of NAPDH, a main substrate of NADPH oxidase, and the ratio of NAPDH to NADP^+^ correspondingly decreased or increased in circ_005077 cell upregulation or downregulation (Fig. 6G∼J), whereas these alterations were reversed by transfection with si-CyPA or OE-p47PHOX (Fig. 6E∼J). These results demonstrate that circ_005077 promotes the interaction between CyPA and p47PHOX to enhance NADPH oxidase activity.

### CyPA and p47PHOX mediated the regulation of circ_005077 in PA-induced myocardial ferroptosis and hypertrophy

**Fig. 7:**
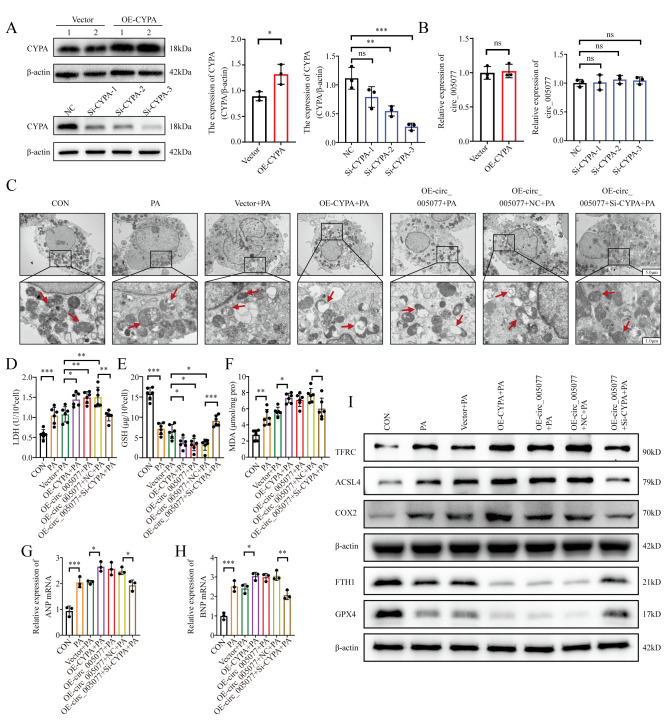
Circ_005077 promoted ferroptosis in H9c2 cells via CyPA. H9c2 cells (C∼I) ) and primary neonatal rat ventricular cardiomyocytes (NRVCs) (G, H) were transfected with vector; OE-CyPA; OE-circ_005077; OE-circ_005077 + NC(co-transfected with OE-circ_005077 and NC); or OE-circ_005077 + Si-CyPA(co-transfected with OE-circ_005077 and Si-CyPA). (**A**) Transfection efficiency of CyPA overexpression and inhibition of CyPA demonstrated by Western blotting. The corresponding quantification was shown. (**B**) The relative expression of circ_005077 in H9c2 cells with inhibition or overexpression of CyPA is shown. (**C**) Representative TEM images of mitochondria in H9C2 cells (1,500×, upper row and magnified views at 6,000, lower row). (**D**) The changes in LDH levels. (**E**) The changes in GSH levels. (**F**) The changes in MDA levels. (**G**) The mRNA expression of ANP in NRVCs was evaluated using qRT-PCR. (**H**) qRT-PCR evaluated the mRNA expression of BNP in NRVCs. (**I**) Western blotting detecting TFRC, ACSL4, COX2, FTH1, and GPX4 protein levels. Data were shown as mean ± SD of at least three independent experiments. *P < 0.05; **P < 0.01; *** *P* < 0.001

**Fig. 8:**
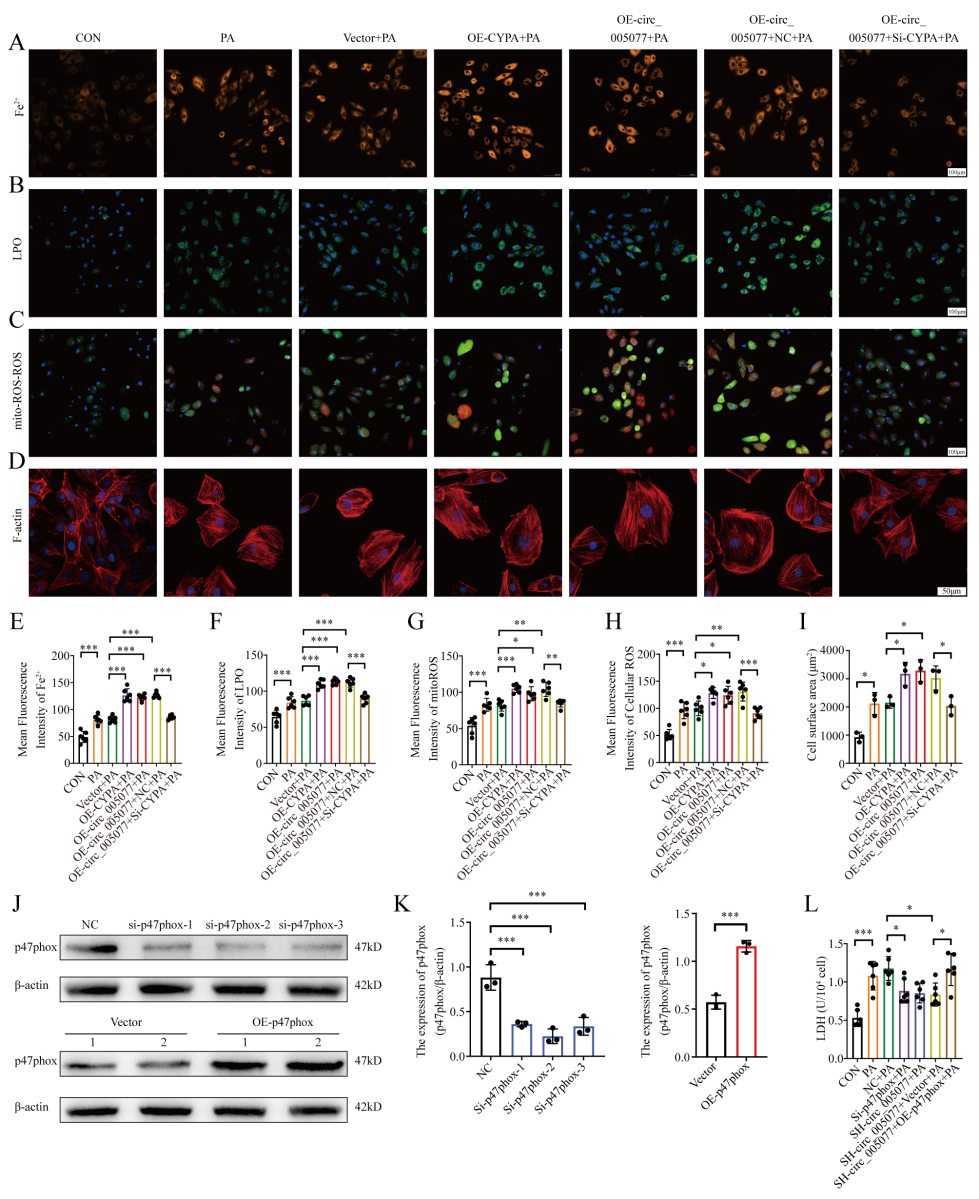
Circ_005077 promoted ferroptosis in H9c2 cells via CyPA/p47phox. (**A**, **E**) Representative immunofluorescent staining and quantitative analysis of ferrous iron (orange, 400× magnification). (**B**, **F**) Representative immunofluorescent staining and quantitative analysis of LPO (green, 400× magnification). (**C**, **G**, **H**) Representative immunofluorescent staining and quantitative analysis of cellular ROS (green,400× magnification) and mitoROS (red, 400× magnification). (**D**, **I**) IF staining (600× magnification) was used to determine the surface area of NRVCs. (**J**, **K**) The transfection efficiency of p47phox overexpression p47phox and inhibition p47phox was demonstrated by Western blotting, and the corresponding quantification was exhibited. (**L**) LDH expression levels. Data were shown as mean ± SD of at least three independent experiments. *P < 0.05; **P < 0.01; *** *P* < 0.001

To confirm the regulatory role of CyPA in PA-induced myocardial ferroptosis and hypertrophy, CyPA was overexpressed and silenced in H9c2 cells transfected with the CyPA plasmid and si-CyPA (Fig. 7A). We found that overexpression and suppression of CyPA have little effect on circ_005077 (Fig. 7B). CyPA overexpression alone can promote PA-induced myocardial ferroptosis and hypertrophy (Fig. 7C-I and Fig. 8A-I), which is similar to overexpression of circ_005077. When CyPA was silenced in H9c2 cells overexpressing circ_005077, the promotion of myocardial ferroptosis and hypertrophy by circ_005077 overexpression was reversed (Fig. 7C-I and Fig. 8A-I), suggesting that CyPA mediates the effect of circ_005077 on PA-induced myocardial ferroptosis and hypertrophy.

**Fig. 9:**
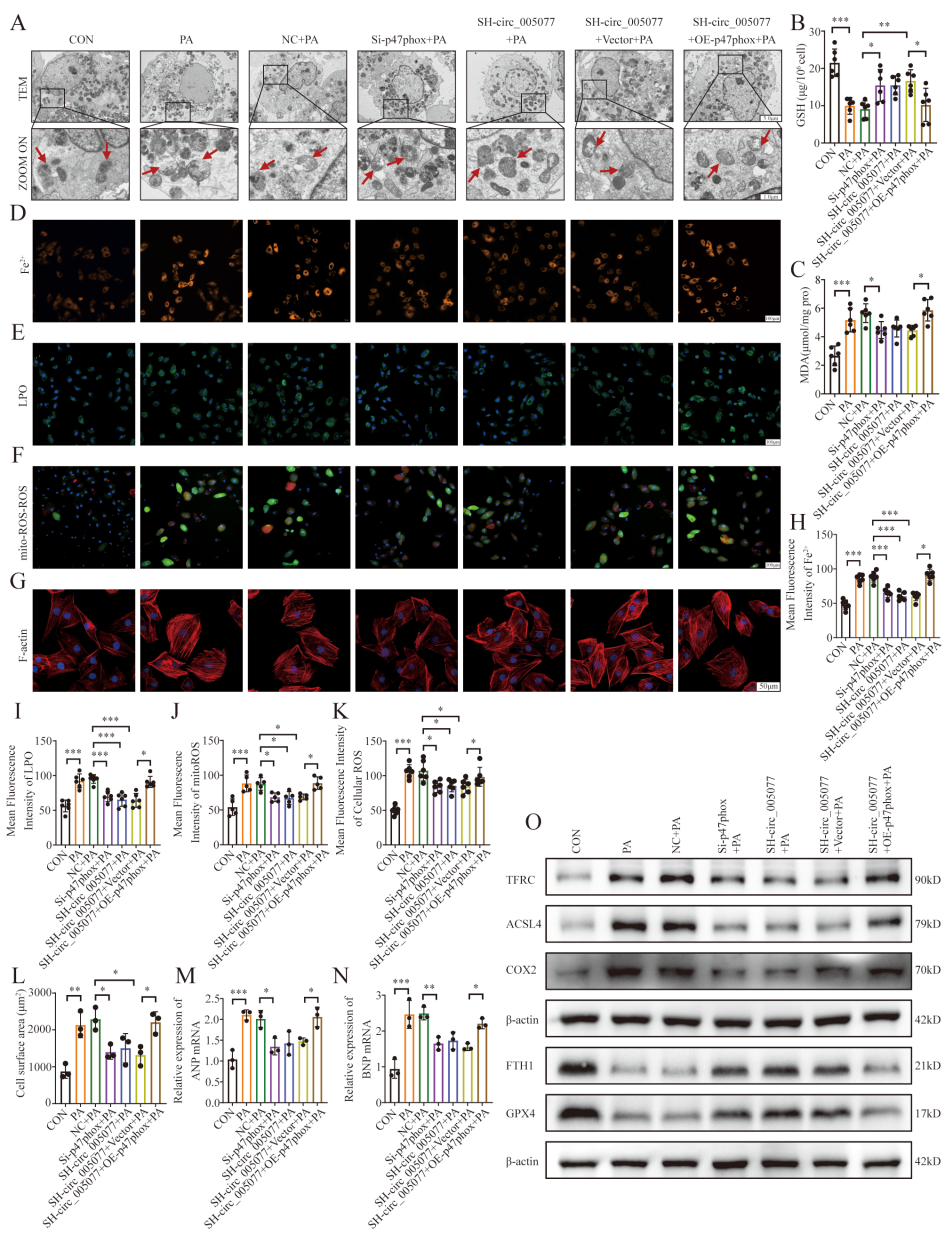
Circ_005077 promoted ferroptosis in H9c2 cells via p47phox. H9c2 cells (A∼F, O) and primary neonatal rat ventricular cardiomyocytes (NRVCs) (G, M, N) were transfected with NC;Si-p47phox;SH-circ_005077;SH-circ_005077 + vector (co-transfected with SH-circ_005077 and vector); or SH-circ_005077 + OE-p47phox(co-transfected with SH-circ_005077 and OE-p47phox). (**A**) Microbiology of mitochondria under electron microscope (1,500×, upper row, and magnified views at 6,000, lower row). (**B**) The expression levels of GSH. (**C**) The MDA expression levels. (**D**, **H**) Ferrous iron representative photos and quantitative analysis (orange, 400× magnification). (**E**, **I**) LPO representative images and quantitative analysis (green, 400× magnification). (**F**, **J**, **K**) MitoROS (red, 400× magnification) and cellular ROS (green, 400× magnification) representative photos and quantitative analysis. (**G**, **L**) F-actin representative images (red,600× magnification) and quantitative analysis of cell surface area. (**M**) Rescue experiments detect the ANP mRNA expression levels in NRVCs by the qPCR assay. (**N**) Rescue experiments detect the BNP mRNA expression levels in NRVCs by the qPCR assay. (**O**) Western blotting detecting TFRC, ACSL4, COX2, FTH1, and GPX4 protein levels. Data were presented as the normalized mean ± SD of at least three independent experiments. *P < 0.05; **P < 0.01; ***P < 0.001. NS, no significance

We also examined the regulation of p47PHOX in PA-induced myocardial ferroptosis and hypertrophy. p47PHOX expression was successfully increased and decreased after transfection with the plasmid p47PHOX plasmid and si-p47PHOX, respectively (Fig. 8J and K). Silencing of p47PHOX alone could repress PA-induced myocardial ferroptosis and hypertrophy, similar to the silencing of circ_005077 (Figs. 8L and 9A∼O). When circ_005077 was silenced in H9c2 cells, repression of myocardial ferroptosis and hypertrophy by circ_005077 silencing was rescued by p47PHOX overexpression (Figs. 8L and 9A∼O), implicating that circ_005077 regulates PA-induced myocardial ferroptosis and hypertrophy through p47PHOX.

### Circ_005077 regulates HFD-induced cardiac dysfunction, myocardial hypertrophy, fibrosis, and ferroptosis in vivo

**Fig. 10:**
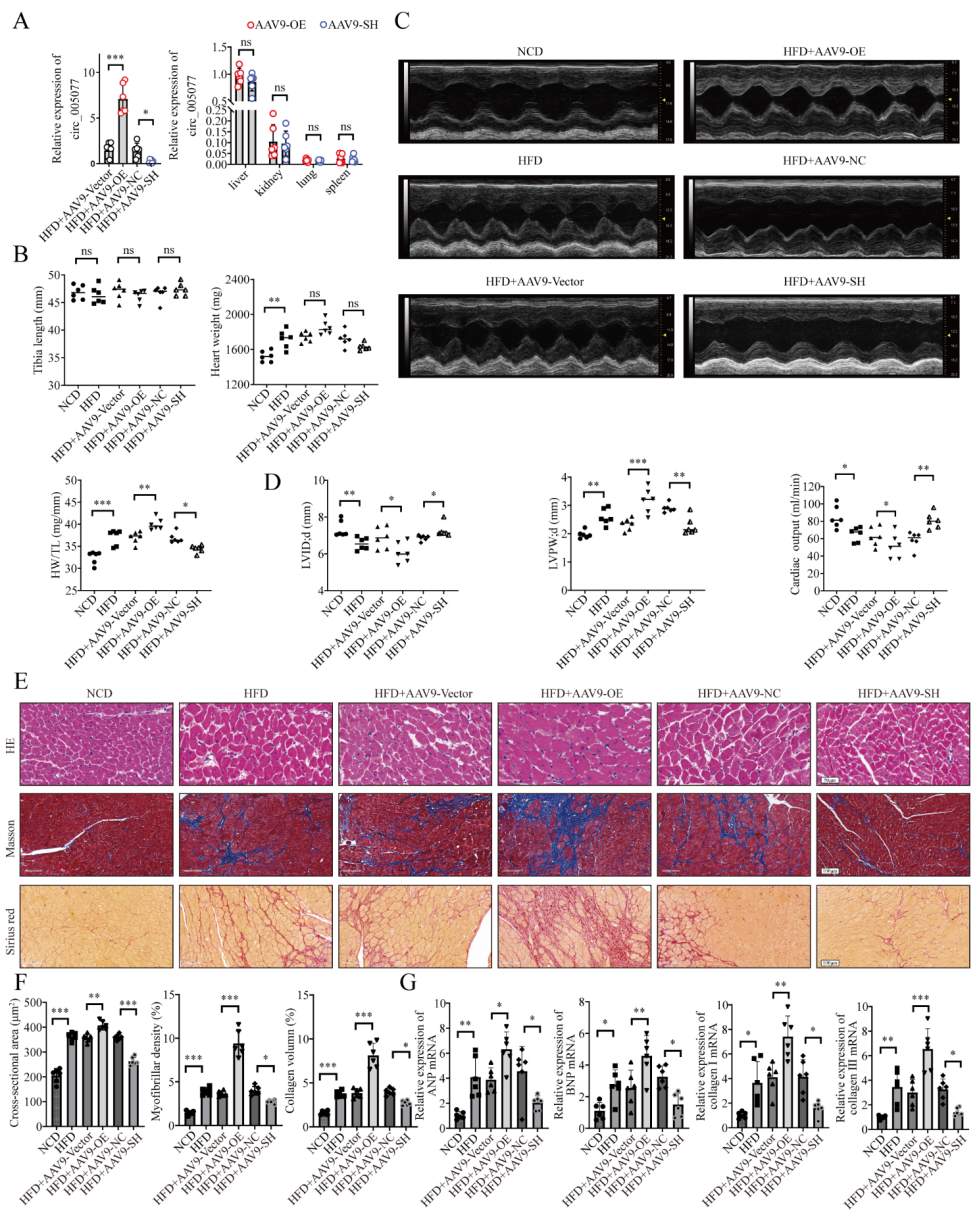
Circ_005077 regulated cardiac hypertrophy and fibrosis of myocardial tissue in vivo. AAV9-OE-circ_005077, AAV9-OE-NC, AAV9-SH-circ_005077, and AAV9-SH-NC were injected individually through the tail vein. (**A**) Relative expression of rno_circ_005077 in the heart, liver, kidney, lung, and spleen after overexpression or silencing of rno_circ_005077 with AAV9 injection. (**B**) Each rat’s heart weight and tibia length and the ratio of heart weight to tibia length (HW/TL) (**C**, **D**) Representative echocardiographic images from the respective groups of rats. Systolic and diastolic function in rat (LVID; d, LVPW; d, CO). (**E**, **F**) HE staining to assess the presence of hypertrophy in cardiomyocytes and statistical results of cross-sectional area (400× magnification); Masson staining to identify myocardial fibrosis and statistical results of the degree of fibrosis (200× magnification); Sirus red staining to determine collagen fiber deposition and statistical results of collagen content (200× magnification). (**G**) qPCR was used to identify hypertrophic marker and fibrosis genes in the indicated groups. AAV9: adeno-associated virus-9; *n* = 6 in each group. The data were displayed as the normalized mean ± SD. *P < 0.05; **P < 0.01; ***P < 0.001

**Fig. 11:**
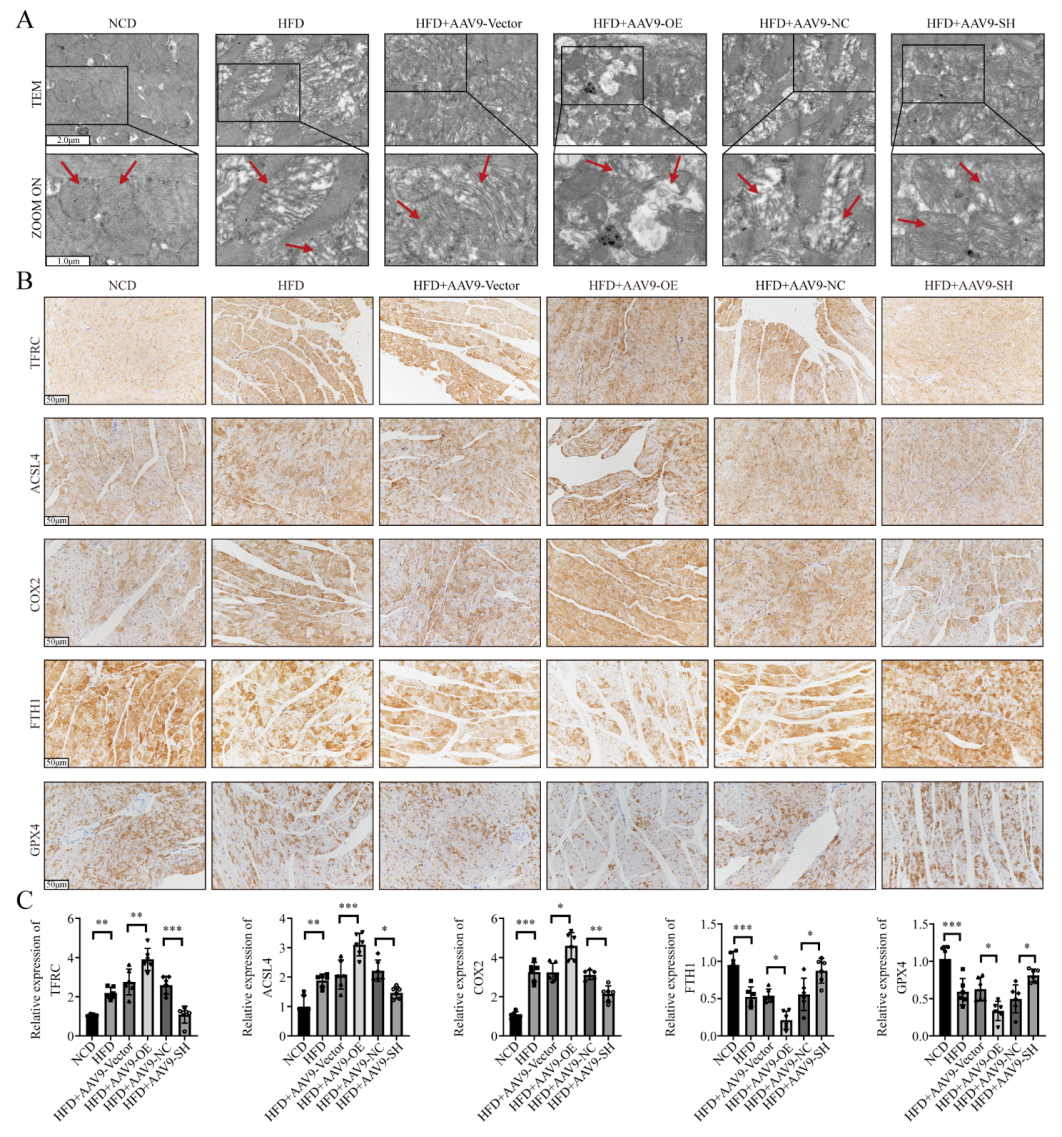
Circ_005077 regulated ferroptosis of myocardial tissue in vivo. AAV9-OE-circ_005077, AAV9-OE-NC, AAV9-SH-circ_005077, and AAV9-SH-NC were injected individually through the tail vein. (**A**) Ultrastructure of the mitochondria and sarcomere using TEM (5,000×, upper row and magnified views at 10,000, lower row) (**B**, **C**) Representative immunohistochemical (IHC) staining of the ferroptosis indicator TFRC, ACSL4, COX2, FTH1 and GPX4 in myocardial tissue (Original magnification: 400×) and quantification of IHC analysis. AAV9: adeno-associated virus-9. Per group, *n* = 6. Data were presented as the normalized mean ± SD.*P < 0.05; **P < 0.01; ***P < 0.001

To test the role of circ_005077 in myocardial lipotoxicity in vivo, the level of circ_005077 was significantly upregulated and downregulated in the rat myocardium by injecting AAV-9 carrying the circ_005077 plasmid and sh-circ_005077 through the tail vein. However, no significant changes were found in the liver, kidney, lung, and spleen (Fig. 10A). When the HFD rats overexpressed circ_005077 were compared to controls, heart weight and the ratio of heart weight to tibia length (HW/TL) significantly increased (Fig. 10B). Meanwhile, two-dimensional ultrasound showed that the left ventricular wall thickness increased while the end-diastolic volume and cardiac output decreased (Fig. 10C and D). Furthermore, overexpression of circ_005077 in HFD rats increased myocardial cross-sectional area, fibrosis, and collagen deposition (Fig. 10E and F), as well as upregulation of hypertrophy-and fibrosis-related genes compared with HFD rats with empty vector (Fig. 10G). In contrast, HFD rats with circ_005077 silencing exhibited opposite phenotypes (Fig. 10B-G). Additionally, we also examined the effect of circ_005077 overexpression or inhibition on HFD-induced ferroptosis in vivo. As shown in Fig. 11, overexpression of circ_005077 in HFD rats promoted myocardial ferroptosis phenotypes. However, inhibition of circ_005077 in the HFD rats repressed the ferroptosis phenotypes.

Taken together, circ_005077 could regulate HFD-induced cardiac dysfunction, myocardial hypertrophy, fibrosis, and ferroptosis in vivo.

## Discussion

Recent studies have reported that circRNA is an important regulator of diabetic cardiomyopathy [43, 44]. For example, circRNA CDR1as promotes cardiomyocyte apoptosis [43] and circRNA DICAR modulates cardiomyocyte pyroptosis in diabetic cardiomyopathy [44]. The lipotoxicity in nonadipose cell in vitro could be induced by PA treatment [45, 46]. Wu L et al. have shown that circ_Tulp4 overexpression alleviates PA-induced beta cell dysfunction [45]. Xu ZX et al. found that circ608 promotes PINK1-mediated mitophagy of liver stellate cells in the present of PA [46]. However, little is known about circRNA’s role in myocardial lipotoxicity. The current study identified circ_005077 as a regulator for PA and HFD-induced myocardial lipotoxicity for the first time, providing a new therapeutic target for myocardial lipotoxicity.

It is widely known that ROS accumulation can cause cell and tissue damage, and that it can also play a regulatory role as a signaling molecule [47]. Oxidative stress is the core mechanism of diabetes [48]. The accumulation of FFA can produce excessive ROS in the condition of diabetes, and ROS can directly damage pancreatic islets β Cells, promoting their apoptosis [49]. In addition, ROS is also involved in the occurrence of diabetes complications, such as endothelial dysfunction, diabetic retinopathy and nephropathy [49]. Given that ferroptosis is characterized by an accumulation of iron-induced lipid ROS [50], overproduction of ROS is an important bridge between ferroptosis and β Cells damage [51], endothelial dysfunction [52], diabetic retinopathy [53] and nephropathy [54]. Similarly, cardiomyocyte is one of the cell types with the highest content of mitochondria [55] and the generation of mitochondrial ROS induced by lipid overload in cardiomyocytes contributes to diabetes or obesity-induced myocardial lipotoxicity [56], therefore, ferroptosis may also be involved in PA or HFD-induced myocardial lipotoxicity. In this study, we found that PA and HFD not only caused the in vitro and in vivo phenotype of myocardial lipotoxicity but also, simultaneously, altered the ferroptosis marker in cardiomyocytes and myocardium, which is similar to the previous report and suggests that PA or HFD is an inducer of ferroptosis [35, 36, 41]. Some researchers have focused on the HFD- or PA-induced ferroptosis mechanism. For instance, Zhao HP et al. have found that the deficiency of the FUN14 domain-containing protein (FUNDC1), a key regulator of mitophagy, increases susceptibility to cardiac dysfunction and remodeling under short-term exposure to HFD through ACSL4-mediated ferroptosis [33]. Meanwhile, Zhu MY et al. have reported that HFD-induced myocardial injury is related to ferritinophagy-mediated ferroptosis activation [35]. Another notable aspect for the regulation of ferroptosis is non-coding RNA, mainly including miRNA, long noncoding RNA and circRNA [57]. For instance, some oncogenic circular RNAs, such as CircPVT1 [58], Circ_0000745 [59], CircGFRA1 [60] and so on, can negatively regulated ferroptosis, thereby leading to tumor growth and metastasis. However, the regulatory mode of ferroptosis has certain cell and tissue specificity [61], the regulation of circRNA in HFD- or PA-induced ferroptosis need to be determined. In the present study, we found that circ_005077 is a novel modulator for ferroptosis, especially for PA or HFD-induced myocardial ferroptosis, providing a new clue to illuminate the regulation of ferroptosis under lipotoxicity condition.

It is one of the important models for circRNA regulation because it can act as microRNA sponges, resulting in increased levels of miRNA targets [22]. Although circ_005077 is predicted to interact with several miRNAs, we could not detect these predicted miRNAs in the product pulled down by circ 005077 (Fig. S5A). This could be because circRNA that functions as miRNA sponges must be highly and widely associated with Argonaute (AGO) proteins; however, circ_005077 was not found to interact with AGO protein in our study (Table S5B).

Compared to the interaction between circRNA and miRNA, circRNA protein models are multiple and more complex [62]. To the best of our knowledge, the interactions of circRNA protein include at least four types of models: (i) circRNAs could act as protein scaffolds to facilitate colocalization and complex formation between enzymes and their substrates (such as phosphatases, transmethylases, and ubiquitin ligases) [63, 64]; (ii) circRNAs may act as protein recruiters to guide proteins to cellular locations [65, 66]; and (iii) circRNAs may reinforce the function of its binding proteins [67]; and (iv) circRNAs could function as protein sponges or decoys by competing with other molecules to bind to specific RBP motifs [68, 69]. CyPA, a protein universally expressed in the cytoplasm, is an important regulator of ROS generation in response to oxidative stress [42]. Furthermore, CyPA can function as a key regulator for regulated cell death [70, 71]. For example, CyPA has been reported to participate in nuclear translocation of AIF during apoptosis [70]. CyPA is also involved in shikonin-induced glioma cell necroptosis and chromatinolysis [71]. RSL3, a ferroptosis inducer, has been shown to cause CyPA release in human proximal tubular HK2 cells [72]. Therefore, we inferred that CyPA is a potential ferroptosis regulator and have choosen it from 47 circ_005077 binding proteins for further study. In our study, circ_005077 interacted with CyPA and inhibited the latter degradation dependent on UBS. We inferred that circ_005077 may function as a protein sponge by blocking the ubiquitination sites of the CyPA protein, protecting it from ubiquitin protease-dependent protein degradation, which is similar to previous reports [68, 69]. NADPH oxidase is the main driving force of oxidative stress during ferroptosis and lipotoxicity, and acts as positive regulator of ferroptosis and lipotoxicity by promotion of ROS production [73, 74]. p47phox is the cytosolic subunit of NADPH oxidase [75]. During activation, p47phox is phosphorylated [75] and translocated to the plasma membrane, coordinating the interaction of the different NADPH oxidase subunits (p67phox, p40phox and p22phox) allowing the formation of an active complex [76]. Growing evidence has shown that CyPA can regulate ROS generation by interacting with p47phox, thereby influencing the activity of NADPH oxidase [42]. In this study, we found that overexpression of circ_005077 in PA-treated cardiomyocytes could promote the interaction of CyPA and p47phox. We believe that one possibility is that circ_005077 can increase the expression level of Cypa in cells, causing the latter to bind more to p47phox induced by PA, assisting in its transfer to the cell membrane and activating NADPH oxidase. Another possibility is that circ_005077 serves as a protein scaffold to facilitate colocalization and complex formation of CyPA and p47phox, thereby improving p47phox function on NADPH oxidase and ROS generation activity. This finding may explain why circ_005077 regulates PA-induced myocardial ferroptosis and lipotoxicity, partly dependent on CyPA and p47phox.

Recent studies have reported that small open reading frames (sORFs) within circRNA can encode a peptide or protein [77]. Circular RNA encoded peptides or proteins are critical players in cancer progression [78] and viral replication [79]. Through bioinformatic analysis (ORFfinder Viewer and IRESite database [80]), we found that circ_005077 has three ORF segments and multiple internal ribosome entry sites (IRESite) (Table S5C), suggesting that circ_005077 may have peptide or protein coding potential. However, our study does not determine the possibility of a peptide- or protein-encoded by circ_005077. This is a limitation that we must acknowledge here.

In this study, we used in vitro and in vivo model induced by PA or HFD, which primarily aims to mimic myocardial lipotoxicity, a common pathophysiological process in the heart of diabetes or obesity, rather than causing a specific disease, such as diabetes cardiomyopathy or obesity cardiomyopathy. Therefore, the model used in this study may not meet the diagnostic criteria of diabetes. Whether the conclusions of this study are completely consistent with those of diabetes cardiomyopathy needs to be verified by diabetes gene knockout animals, such as diabetic (db/db) mice.

**Fig. 12:**
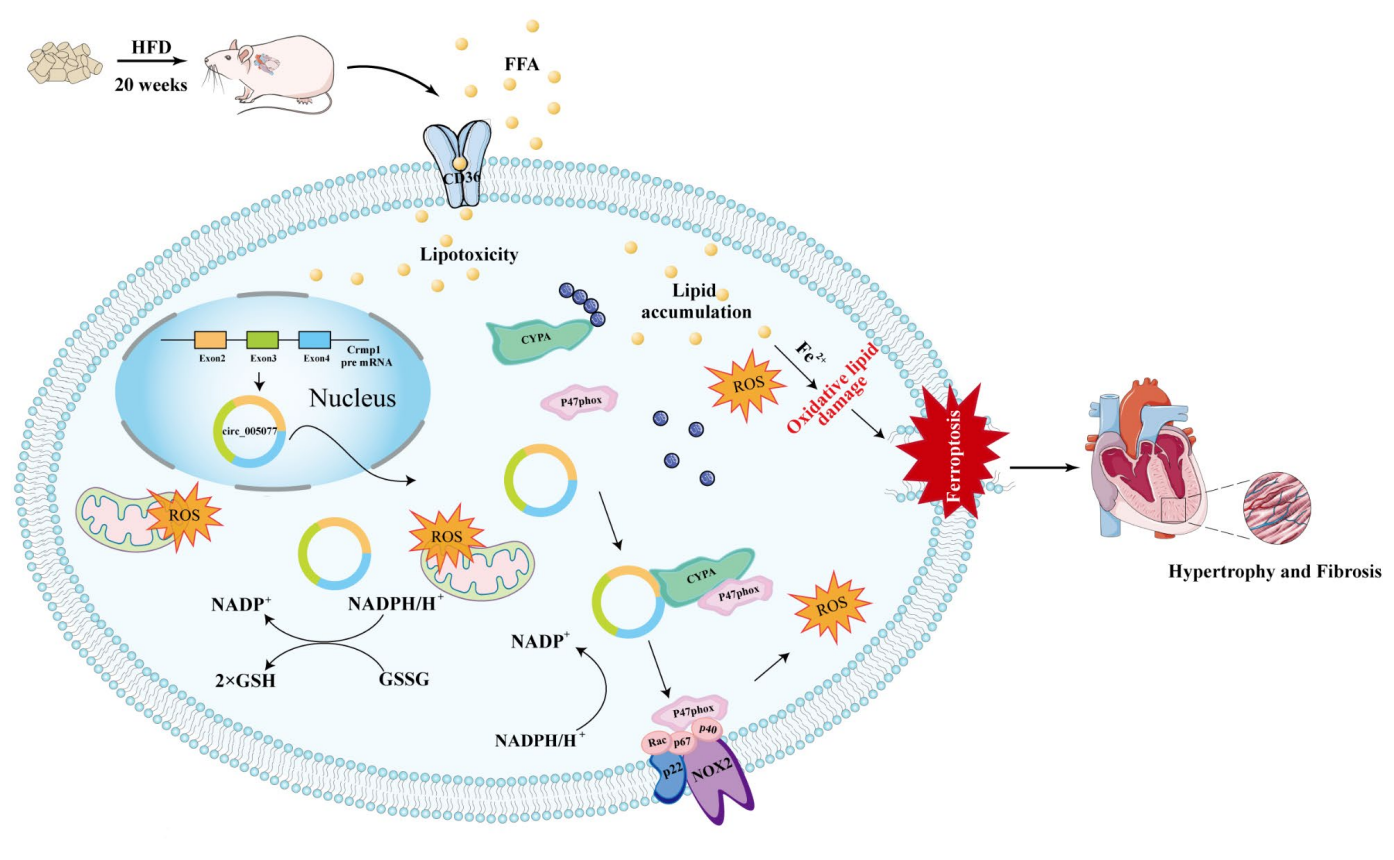
A schematic outlining the proposed mechanisms that circ_005077 accelerates myocardial lipotoxicity induced by high-fat diet via CyPA/p47PHOX mediated ferroptosis

Finally, we show that circ_005077 can directly interact with CyPA and increase NADPH oxidase activity and ROS content by promoting the interaction of CyPA and p47phox, thereby regulating PA/HFD-induced myocardial ferroptosis (Fig. 12). Our results provide new insights into the mechanisms of myocardial lipotoxicity, potentially leading to the identification of a novel therapeutic target for the treatment of myocardial lipotoxicity in the future.

### Electronic supplementary material

Below is the link to the electronic supplementary material.

Supplementary Material 1

## Data Availability

No datasets were generated or analysed during the current study.
