# Supplementary material for: Circ_005077 accelerates myocardial lipotoxicity induced by high-fat diet via CyPA/p47PHOX mediated ferroptosis
Source: Cardiovasc Diabetol. 2024 Apr 15;23:129. doi: 10.1186/s12933-024-02204-3 (PMC11020354; doi:10.1186/s12933-024-02204-3)

Figure S1:Biochemical blood analyses in modular rats.


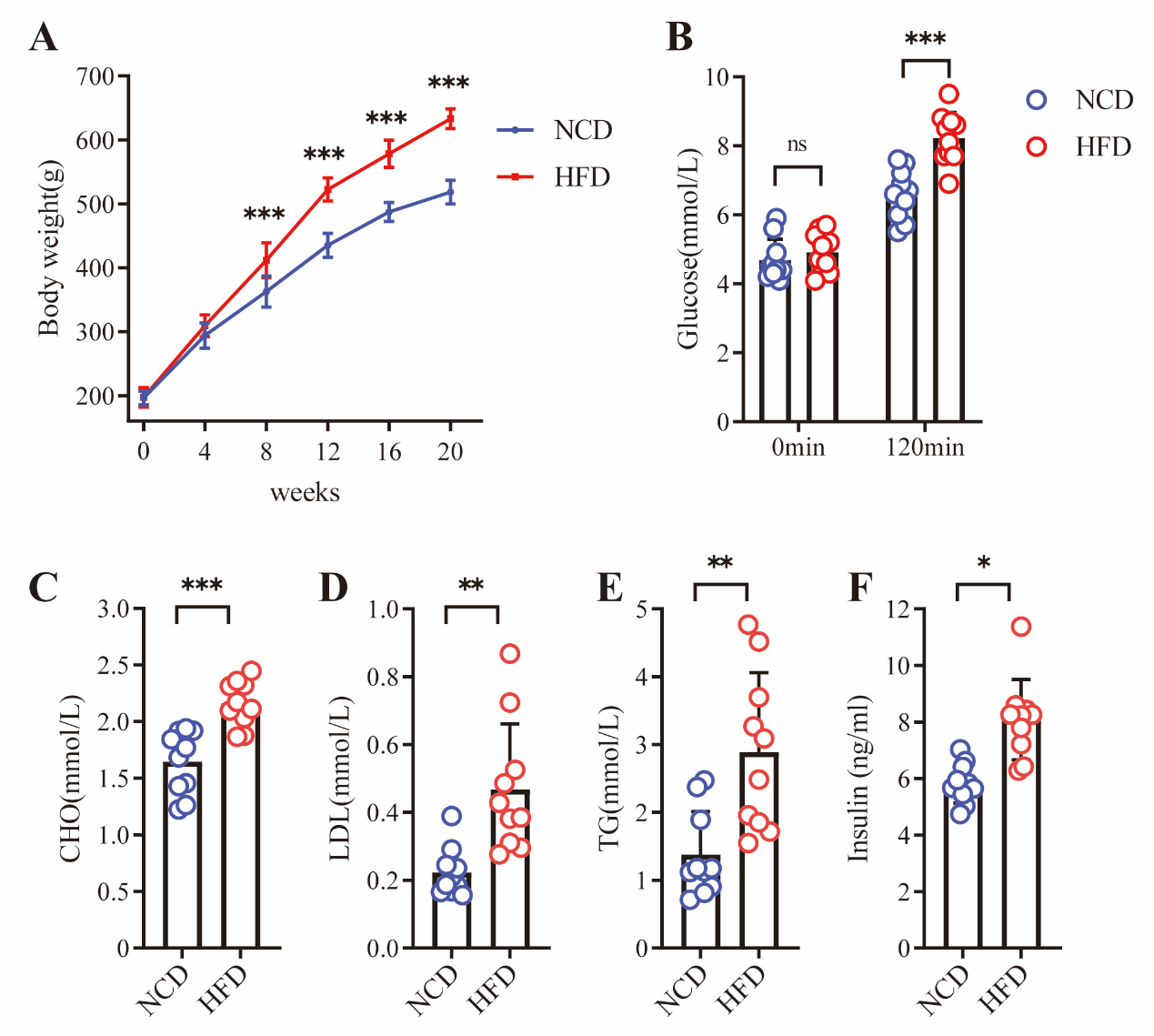


A. Body weight. B. The fasting and OGTT Glucose Tolerance Test glucose levels. C. Serum cholesterol levels. D. Serum low-density lipoprotein levels. E. Serum triglyceride levels. F. Serum insulin levels. NCD: normal diet, HFD: high-fat diet. Data were shown as mean ± s.d. * p < 0.05; ** p < 0.01; ***P < 0.001. n = 10 in each group.

Table S1A.Baseline data sheet of echocardiography in modular rats(n=10)

Table S1B:The top twenty circRNAs with significant fold changes both in upregulated and downregulated groups(n=10)

Table S1C:All the upregulated circRNAs with significant fold changes(n=21).


Table S1D:All the downregulated circRNAs with significant fold changes(n=186).

Table S1E: The primer sequences used are as follows.

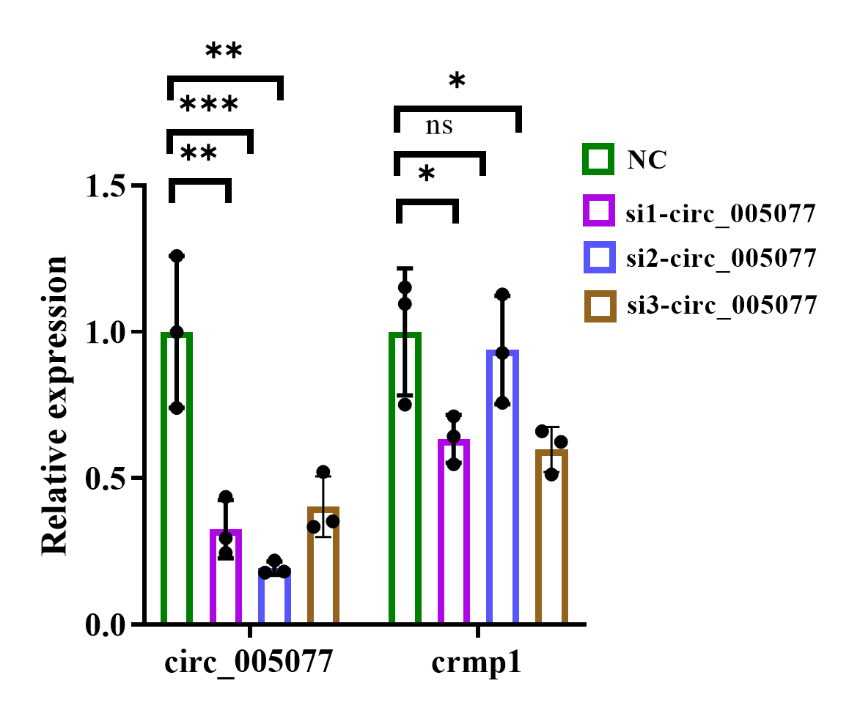
Figure S2: Knockdown efficiency assay of circ_005077 and effect on crmp1.

Data were shown as mean ± s.d. of three independent experiments. *P < 0.05; **P < 0.01; *** P < 0.001;NS,no significance.

Table S2: All the siRNA sequences used as followed.

Figure S4: Quantification of TFRC, ACSL4, COX2, FTH1, and GPX4 protein levels.


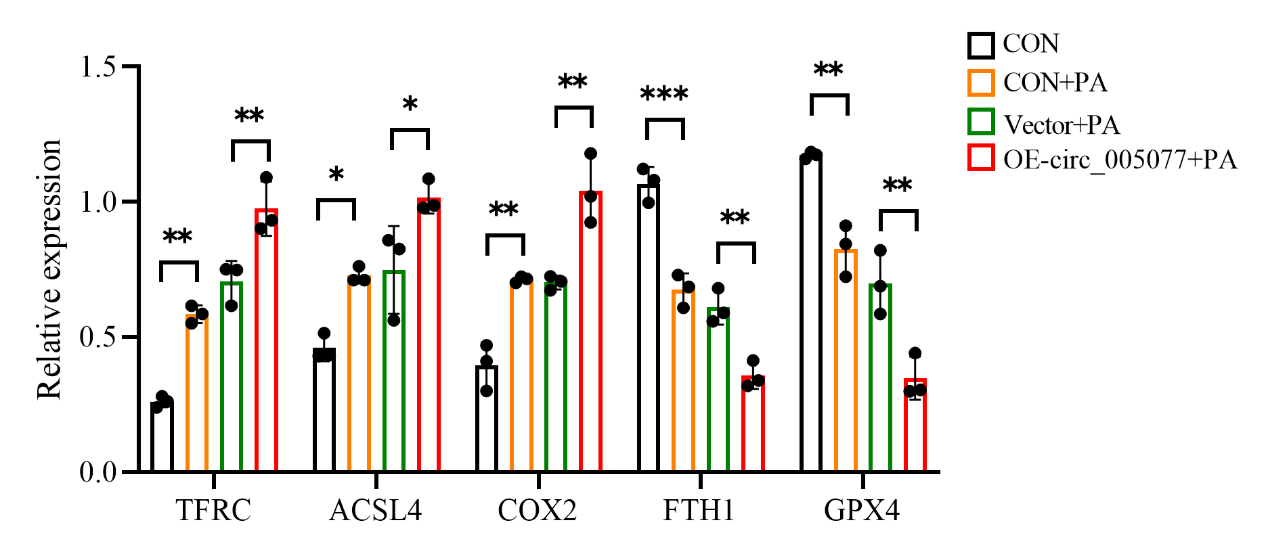


Data were shown as mean ± s.d. of three independent experiments. *P < 0.05; **P < 0.01; *** P < 0.001.


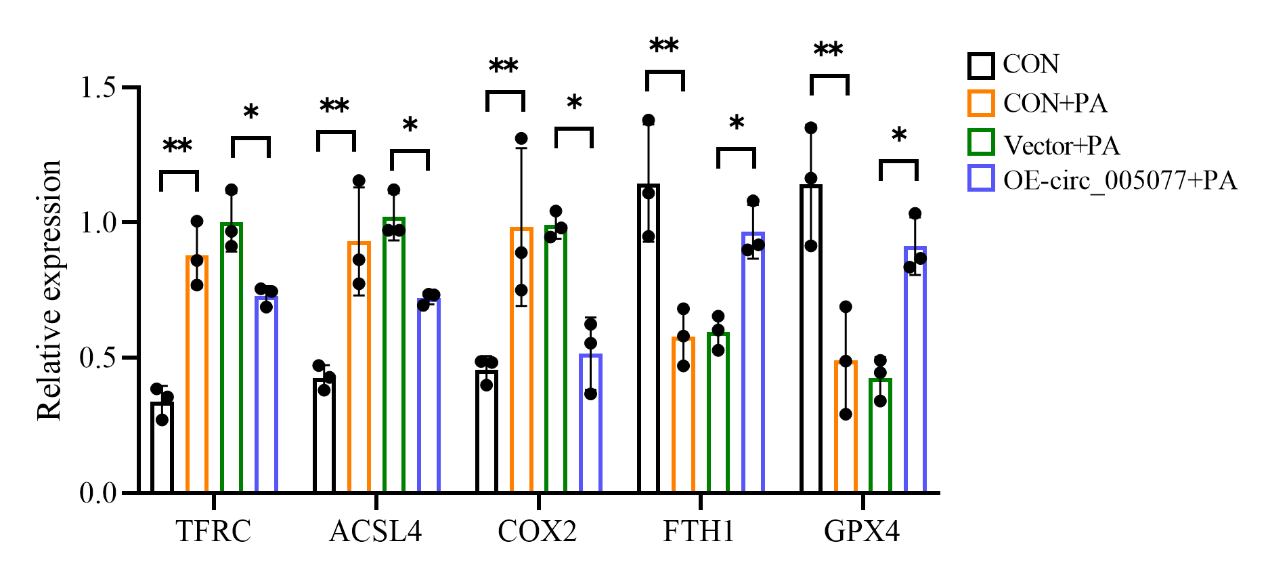


Data were shown as mean ± s.d. of three independent experiments. *P < 0.05; **P < 0.01; *** P < 0.001.

Table S5A. miRNAs predicted by online databases that can be combined with rno_circ_005077.

Figure S5A: 10 miRNAs were selected to verify binding to circ_005077.


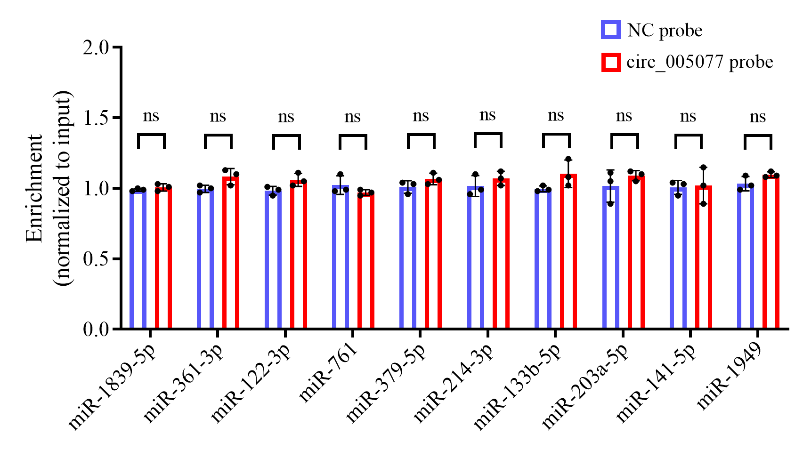


Data were shown as mean ± s.d. of three independent experiments. NS,no significance.

Table S5B. rno_circ_005077 specific binding proteins identified by ChIRP-MS.


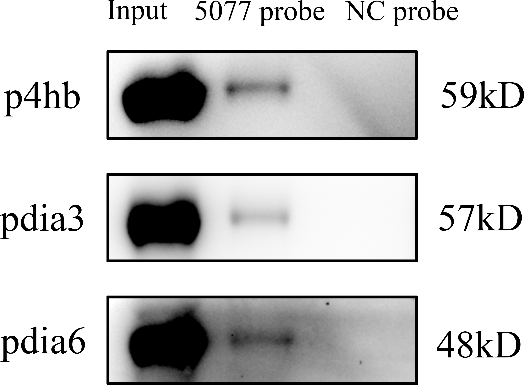
Figure S5B: Specific proteins verified by CHIRP-WB to bind to circ_005077.

Table S5C: Circ_005077 potential functional regions encoding polypeptides.


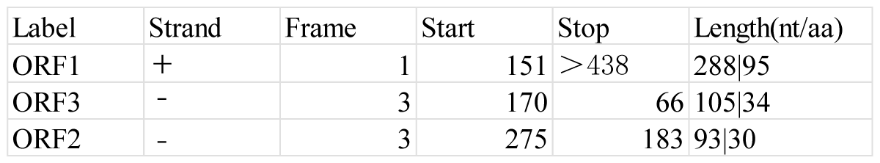


Table S5D: The probe sequences used are as follows.

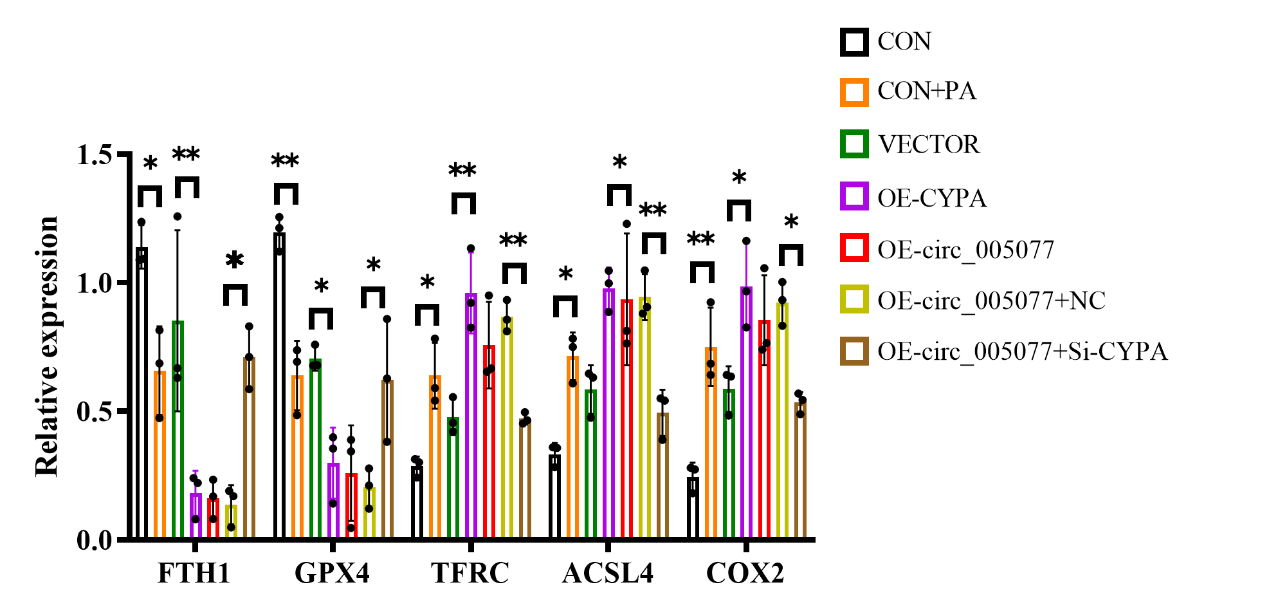
Figure S7: Quantification of TFRC, ACSL4, COX2, FTH1, and GPX4 protein levels.

Data were shown as mean ± s.d. of three independent experiments. *P < 0.05; **P < 0.01.

Figure S9 : Quantification of TFRC, ACSL4, COX2, FTH1, and GPX4 protein levels.


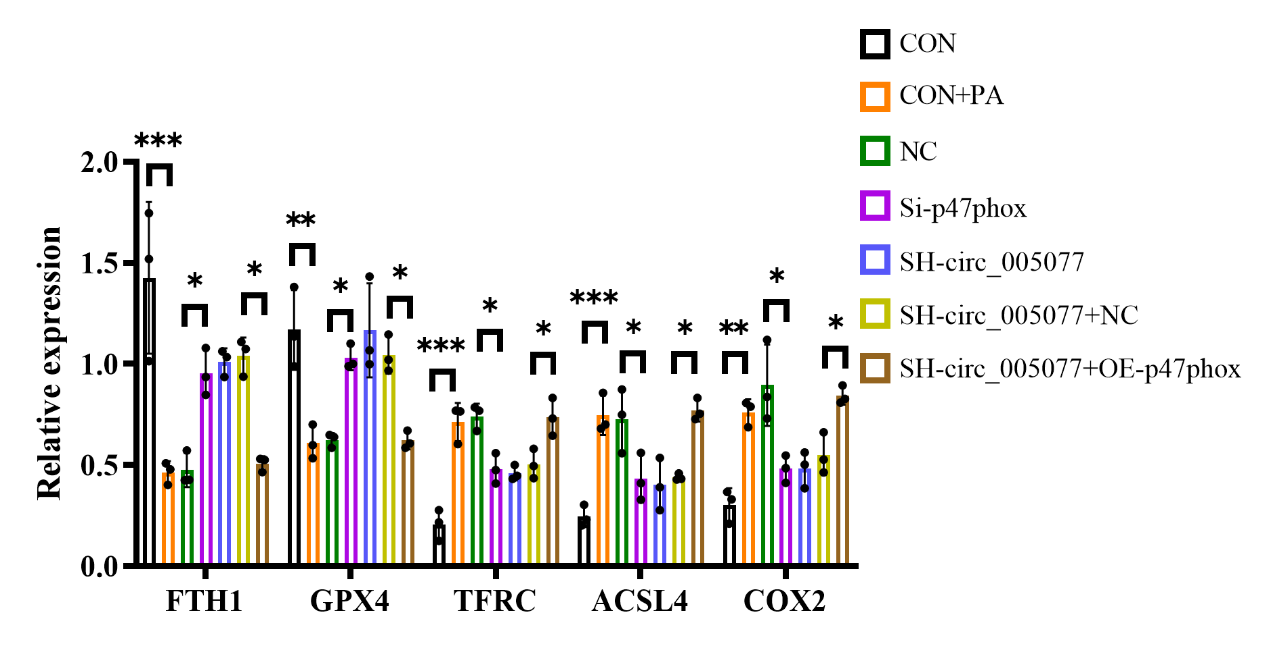


Data were shown as mean ± s.d. of three independent experiments. *P < 0.05; **P < 0.01; *** P < 0.001..

Table S10.Baseline data sheets of echocardiography in every group rats(n=6)

Figure S10: Effect of transfection of rat myocardium shown by fluorescence.

Transfection heart Transfection kidney Transfection lung


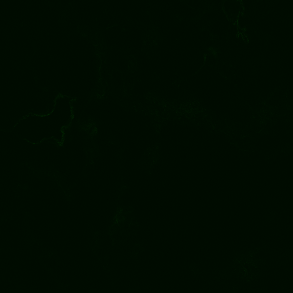

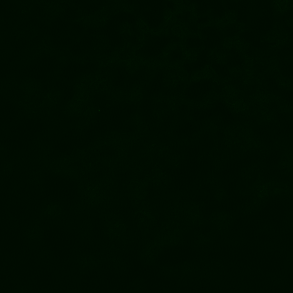

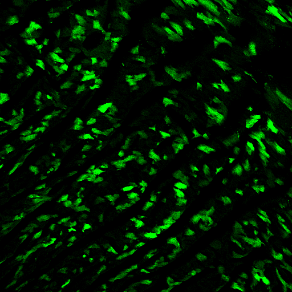


Normal heart Transfection liver Transfection spleen


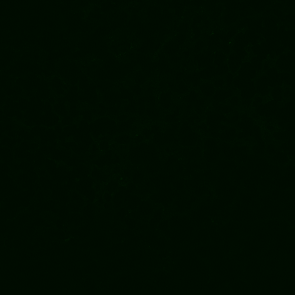

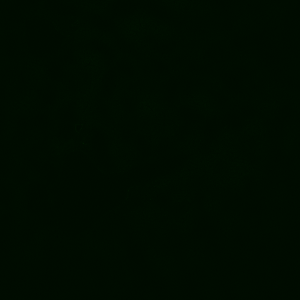

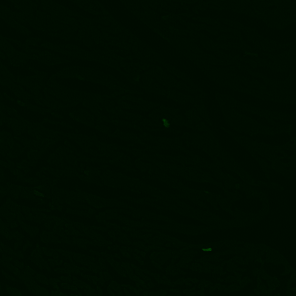


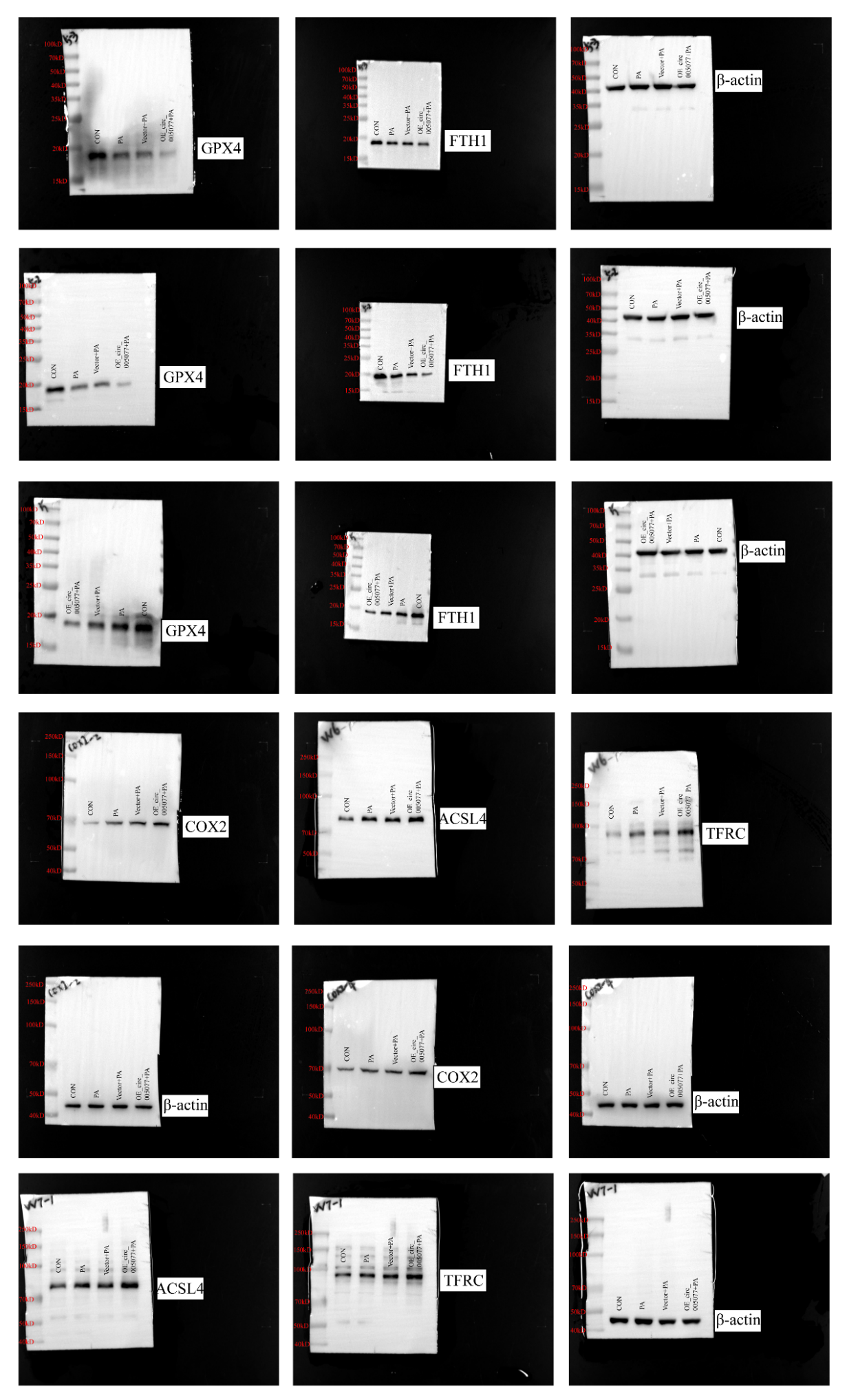
Figure S12: Pictures of western blot full film.


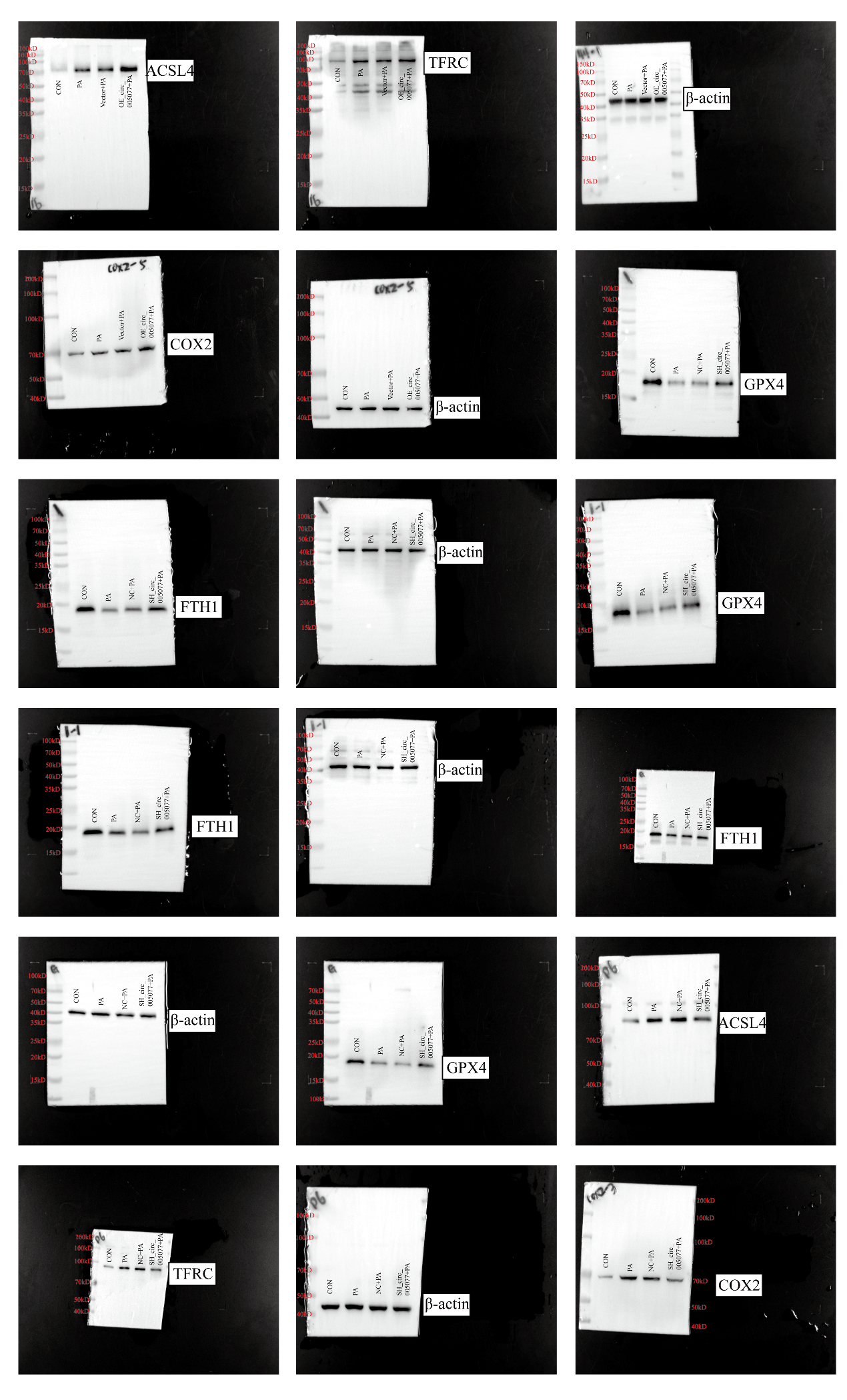


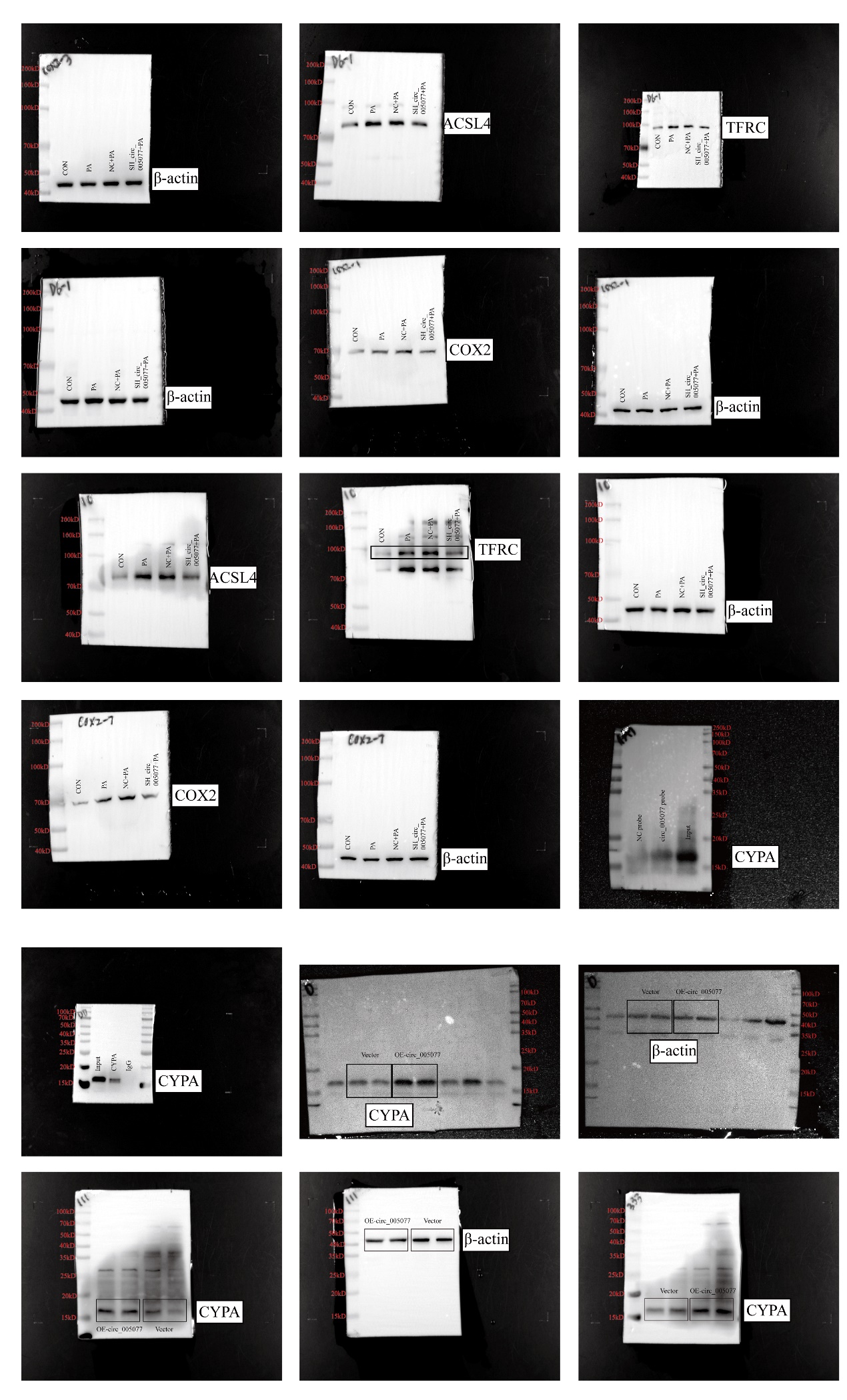


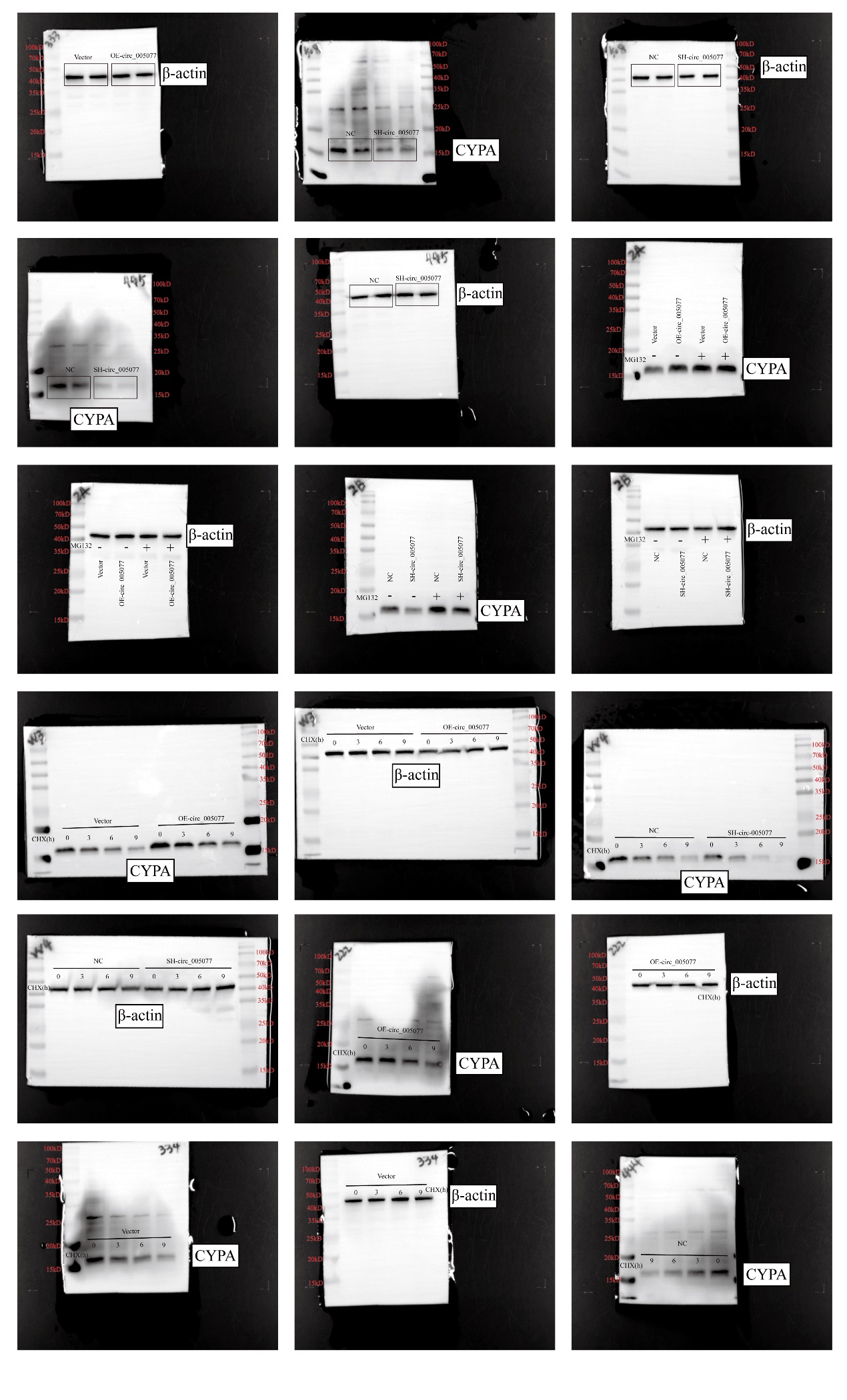


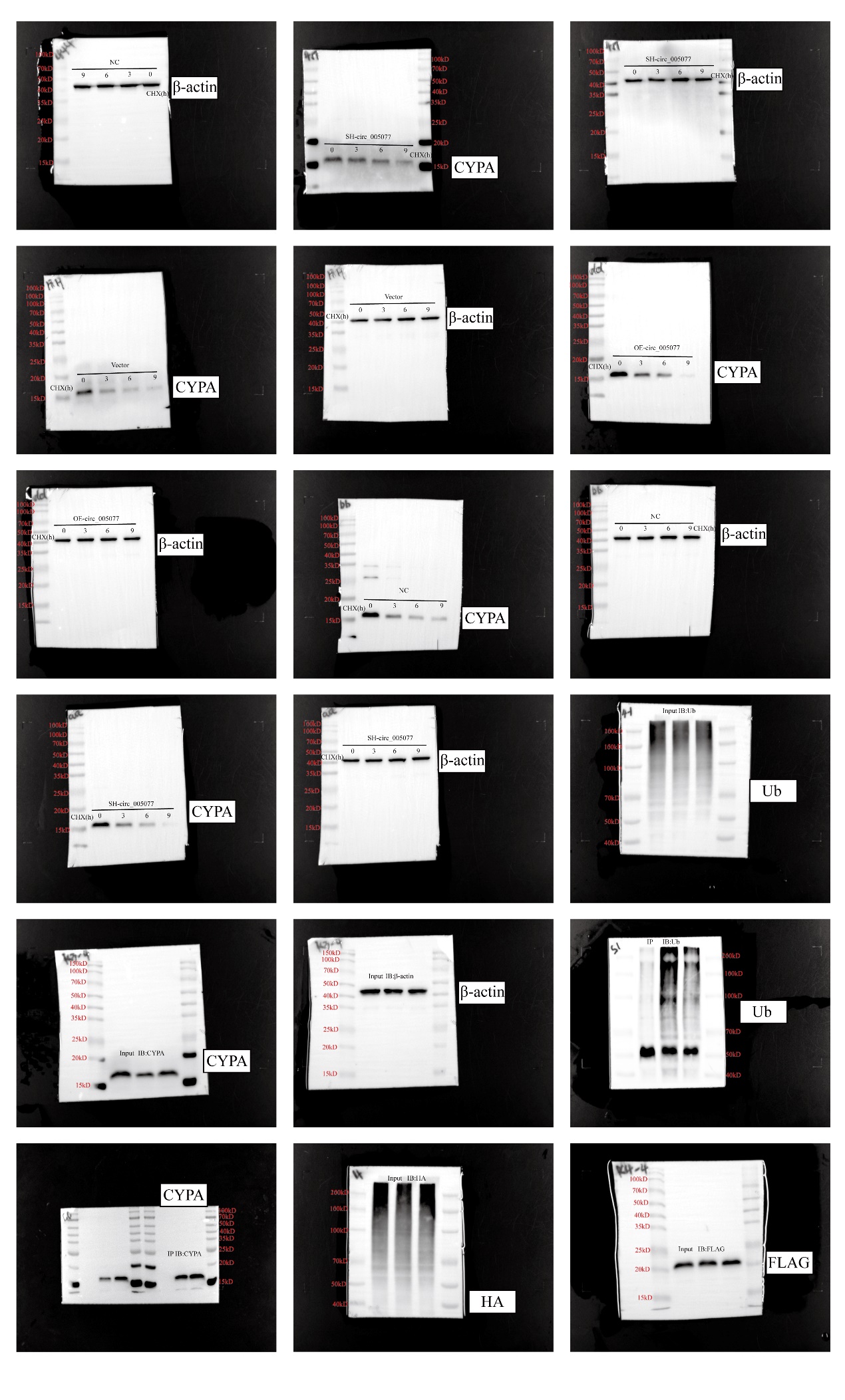


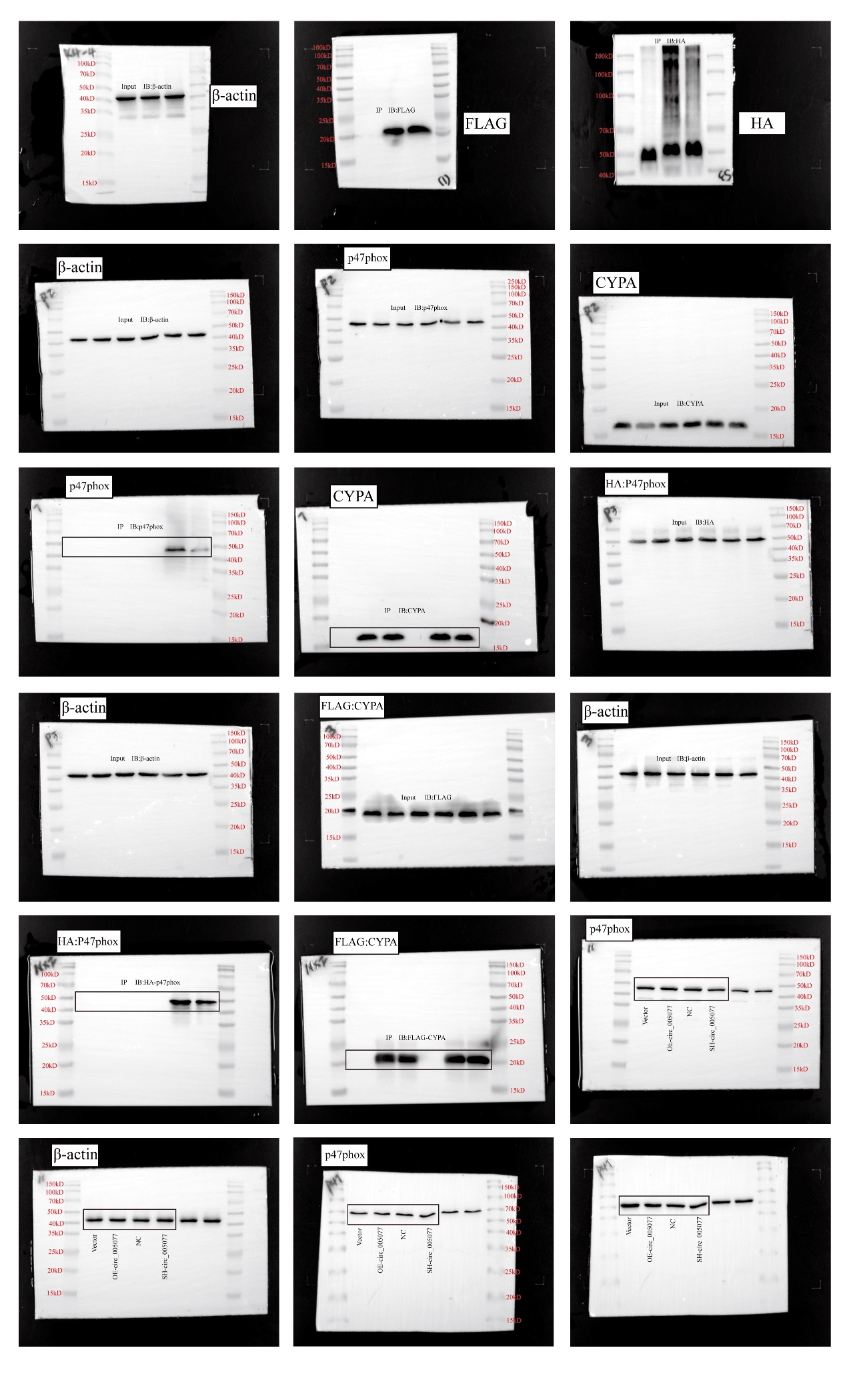


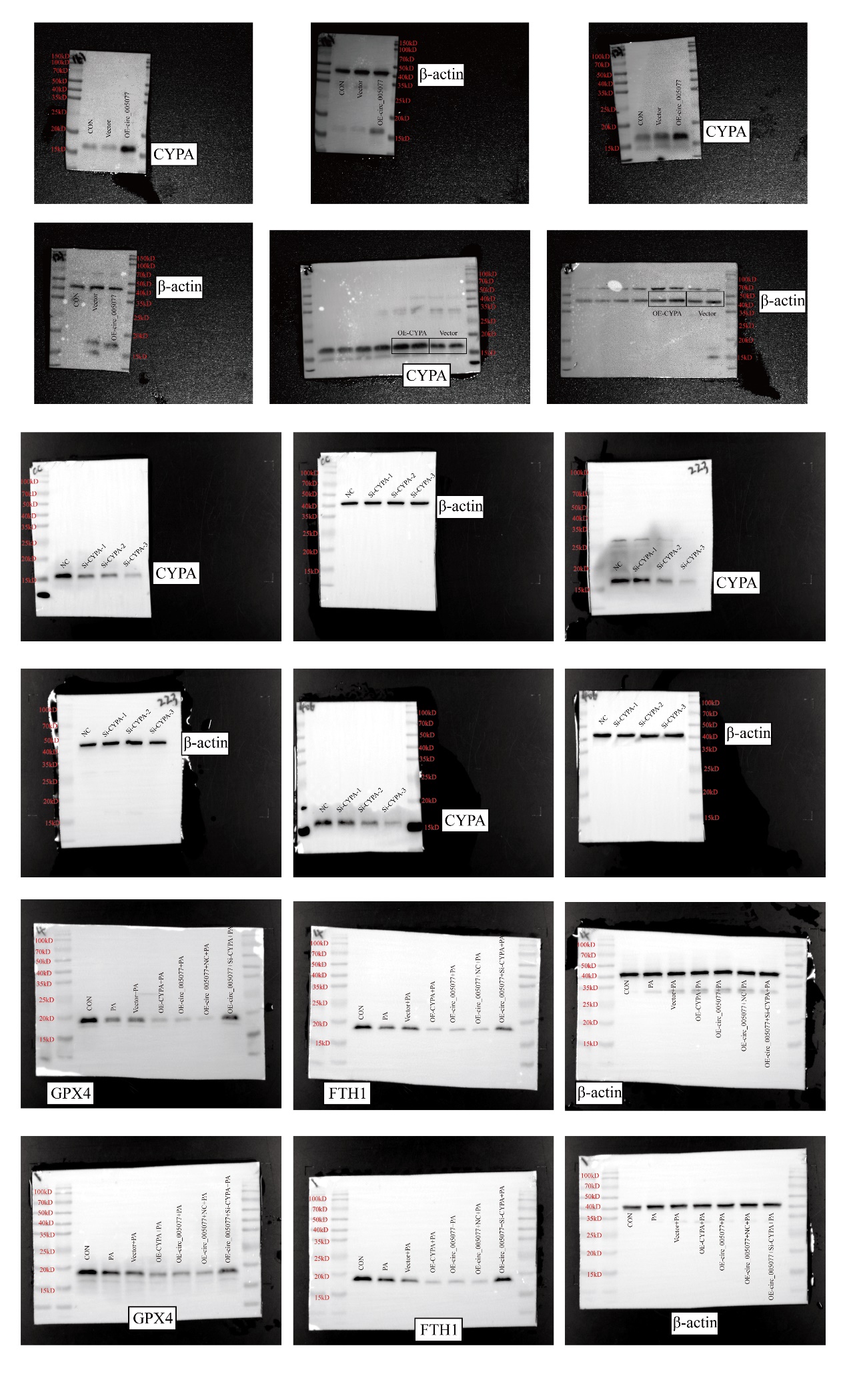


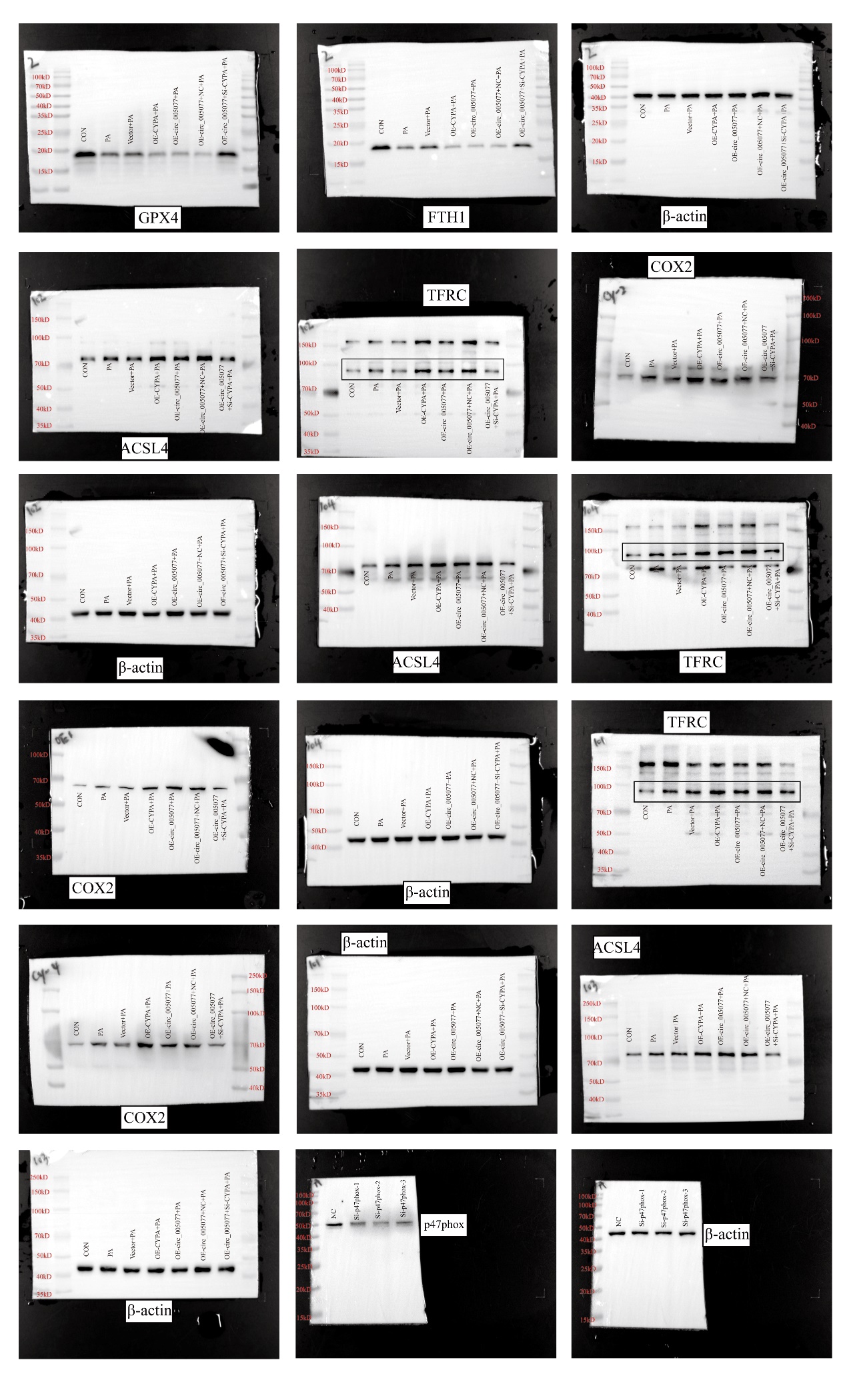


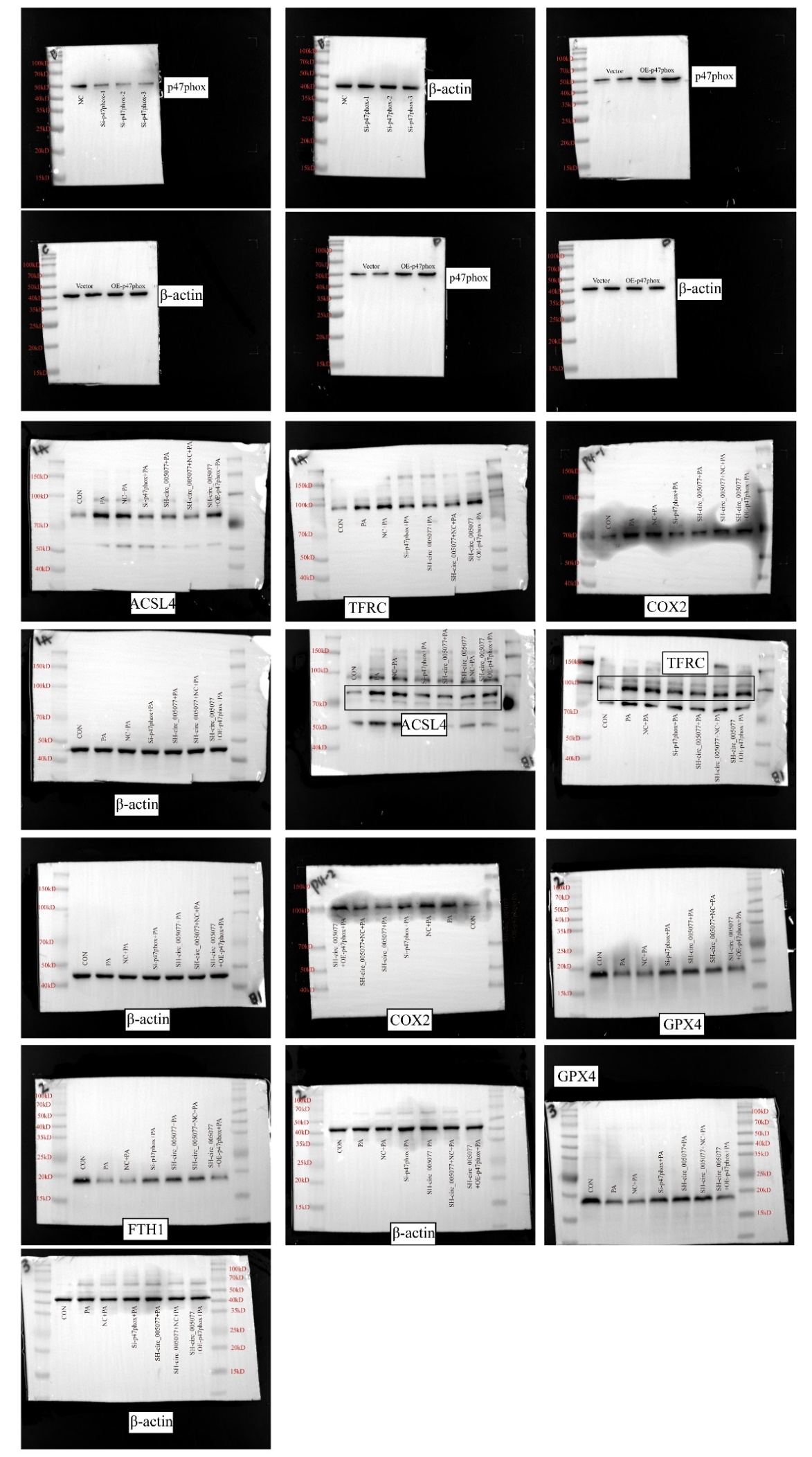

Supplement: Supplementary file 1 — Supplementary Material 1 [file 12933_2024_2204_MOESM1_ESM.docx]
